# Biological Effects in Cancer Cells of Mono- and Bidentate Conjugation of Cisplatin on PAMAM Dendrimers: A Comparative Study

**DOI:** 10.3390/pharmaceutics15020689

**Published:** 2023-02-17

**Authors:** Cláudia Camacho, Dina Maciel, Helena Tomás, João Rodrigues

**Affiliations:** CQM–Centro de Química da Madeira, Molecular Materials Research Group (MMRG), Campus da Penteada, Universidade da Madeira, 9000-390 Funchal, Portugal

**Keywords:** cisplatin, anionic PAMAM dendrimer, monodentate, bidentate, anticancer, metallodendrimer

## Abstract

Cisplatin (*cis*-diamminedichloroplatinum(II)) is a potent chemotherapeutic agent commonly used to treat cancer. However, its use also leads to serious side effects, such as nephrotoxicity, ototoxicity, and cardiotoxicity, which limit the dose that can be safely administered to patients. To minimize these problems, dendrimers may be used as carriers for cisplatin through the coordination of their terminal functional groups to platinum. Here, cisplatin was conjugated to half-generation anionic PAMAM dendrimers in mono- and bidentate forms, and their biological effects were assessed in vitro. After preparation and characterization of the metallodendrimers, their cytotoxicity was evaluated against several cancer cell lines (A2780, A2780cisR, MCF-7, and CACO-2 cells) and a non-cancer cell line (BJ cells). The results showed that all the metallodendrimers were cytotoxic and that the cytotoxicity level depended on the cell line and the type of coordination mode (mono- or bidentate). Although, in this study, a correlation between dendrimer generation (number of carried metallic fragments) and cytotoxicity could not be completely established, the monodentate coordination form of cisplatin resulted in lower IC_50_ values, thus revealing a more accessible cisplatin release from the dendritic scaffold. Moreover, most of the metallodendrimers were more potent than the cisplatin, especially for the A2780 and A2780cisR cell lines, which showed higher selectivity than for non-cancer cells (BJ cells). The monodentate G0.5COO(Pt(NH_3_)_2_Cl)_8_ and G2.5COO(Pt(NH_3_)_2_Cl)_32_ metallodendrimers, as well as the bidentate G2.5COO(Pt(NH_3_)_2_)_16_ metallodendrimer, were even more active towards the cisplatin-resistant cell line (A2780cisR cells) than the correspondent cisplatin-sensitive one (A2780 cells). Finally, the effect of the metallodendrimers on the hemolysis of human erythrocytes was neglectable, and metallodendrimers’ interaction with calf thymus DNA seemed to be stronger than that of free cisplatin.

## 1. Introduction

Cisplatin was the first metal-based anti-cancer drug approved for clinical use and is now applied in treating several cancer types [1,2]. Its pharmacological target is the DNA, which forms stable complexes through intra- and inter-strand crosslinks, modifying its structure, preventing its replication and transcription, and, thus, inducing cellular apoptosis. The intermediates that react with DNA are the aquated cationic complexes *cis*-[PtCl(H_2_O)(NH_3_)_2_]^+^ and *cis*-[[Pt(H_2_O)_2_(NH_3_)_2_]^2+^ that are formed through the hydrolysis of cisplatin inside the cells [3,4,5,6]. These complexes are very reactive and can interact with nucleophiles such as DNA or other biomolecules in the cytoplasm [7]. Moreover, the cisplatin used in clinics presents some limitations due to the adverse secondary effects (e.g., nephrotoxicity, ototoxicity, cardiotoxicity), lack of selectivity, possible development of drug resistance, and low solubility [8,9,10,11]. To overcome these unwanted issues, the use of nanocarriers to deliver drugs has been presented as a helpful strategy [9,11]. For instance, different types of organic (e.g., micelles, liposomes, and dendrimers), inorganic (e.g., carbon nanotubes, gold NPs), and hybrid nanosystems (e.g., nanoscale coordination polymers) were used to deliver cisplatin, with some of them reaching clinical trials [12]. Between them, Lipoplatin (NC-604 or Nanoplatin^TM^), a liposomal polymer-metal complex formed between cisplatin and poly(ethylene glycol)-poly(glutamic acid) block copolymers (PEG-P(Glu)) [2], deserves to be highlighted because it has reached phase 3 in clinical trials with interesting results in different types of cancers, such as ovarian [13] and breast cancer [14] among others. However, the production of cisplatin liposome formulations has some constraints concerning low lipophilicity and water solubility, which reduces the effectiveness of encapsulation, the low drug/lipid ratio, and cytotoxicity, contributing to cisplatin cancer cell resistance [15,16]. In this sense, dendrimers are at the forefront, as they have shown potential for use as drug delivery systems [11,17]. Dendrimers are monodisperse polymers possessing a central core around which the monomers are bound, layer upon layer (forming different generations), giving rise to structures of various sizes and molecular weights [18]. A unique feature of dendrimers is that they are multivalent—that is, they have many terminal chemical groups capable of being chemically functionalized. Due to this and other properties (e.g., solubility in water), dendrimers have been studied as an alternative in several biomedical applications, especially in drug delivery [19,20]. These hyperbranched systems, particularly the PAMAM dendrimers family, are among the most selected types of dendrimers to study and evaluate the capacity to deliver cisplatin and its derivatives in cells [21,22].

Although there is extensive literature on the mono- and bidentate coordination of cisplatin in different drug carriers (e.g., in linear and non-linear polymers) [21,23,24], the study of the monodentate coordination of cisplatin in dendrimers via carboxylic acid/carboxylate terminal groups and its impact on the cells (e.g., in the cytotoxicity) remains to be performed [12,25]. In line with our team’s expertise in the use of dendrimers for biomedical applications [26,27,28,29,30,31], the goal of this study was to, for the first time, assess the effect of the coordination mode of the ligand/metal and the generation of the dendrimer (number of metal fragments it carries) on the delivery of cisplatin to cancer cells using anionic poly(amidoamine) (PAMAM) dendrimers, with carboxylate groups at their periphery. In more detail, several generations of anionic PAMAM dendrimers (the half generations G0.5 to G3.5) were functionalized, with cisplatin in mono- and bidentate forms and characterized with nuclear magnetic resonance spectroscopy (NMR), mass spectrometry (MS), Fourier-transform infrared spectroscopy (FTIR), ultraviolet-visible spectroscopy (UV-Vis), and fluorescence spectroscopy. Then, their cytotoxic behavior was evaluated against a set of cancer cell lines that are representative of different cancer types (A2780, A2780cisR, MCF-7, and CACO-2 cells), as well as a model of a non-cancer cell line (BJ cells). In summary, this study aimed to use the anionic PAMAM dendrimers as metallodrug carriers to evaluate the impact of the ligand/metal coordination process mode (mono- and bidentate forms of cisplatin) and the dendrimer generation effect (number of carried metallic fragments) in cancer cells. 

## 2. Materials and Methods

### 2.1. Materials

All the chemicals were used as received, except for the anionic half-generation PAMAM dendrimers (0.5–3.5) purified through dialysis to eliminate impurities. Anionic half-generation PAMAM dendrimers G0.5 (19.19 *w*/*w* % in methanol), G1.5 (20.03 *w*/*w* % in methanol), G2.5 (9.98% *w*/*w* in methanol) or G2.5 (3.43 *w*/*w* % in water), and G3.5 (10.04 *w*/*w* % in methanol) with an ethylenediamine core were acquired from Dendritech^®^ Inc. Cis-diamminedichloroplatinum (II) (cisplatin 99.99%) was obtained from Acros Organics. Deoxyribonucleic acid sodium salt from the calf thymus (CT-DNA) was purchased from Sigma-Aldrich. The other reagents used in the synthesis were obtained from Acros organics or Fisher Scientific. Unless otherwise stated, all the reagents and media solutions used for cell culture were purchased from Life Technologies (Thermo Fischer Scientific). Dialysis membranes (MW: 100–500 Da, 1000 Da, 2000 Da, 3500 Da, 6000–8000 Da) were bought from SpectrumLabs. The ultrapure water (UPW) used in the experiments was produced with a Milli-Q Direct 8 Water Purification System with a resistivity higher than 18.2 MΩ·cm. For the hemotoxicity assays, healthy human blood was supplied by Hospital Dr. Nélio Mendonça (SESARAM) under the existent collaboration between the University of Madeira/Centro de Química da Madeira and the SESARAM hematology service.

### 2.2. Synthesis and Characterization

By adapting previous methodologies [32,33,34], two different procedures were implemented to conjugate cisplatin to the anionic PAMAM dendrimers. Firstly, cisplatin was directly conjugated to the anionic PAMAM dendrimer in a monodentate form (see Section 2.2.1). Secondly, silver nitrate was used to remove the two chloride groups from cisplatin before conjugating the anionic PAMAM dendrimer in a bidentate form (see Section 2.2.2). The ^1^H, ^13^C, and ^1^⁹⁵Pt-NMR characterization were performed in a Bruker Avance II+ UltraShield^TM^ 400 Plus Ultra Long Hold NMR spectrometer equipment at room temperature, using a 5 mm BBO multinuclear probe. The mass spectrometry analysis was conducted using the electrospray ionization technique (ESI), in positive mode, with the QTOF hybrid analyzer model MAXIS II from Bruker at the Mass Spectrometry Unit at Interdepartmental Investigation Service at Universidad Autónoma de Madrid, Spain, and with the Bruker Autoflex maX MALDI TOF/TOF Mass Spectrometer at Centro de Química da Madeira in University of Madeira. Accordingly, the monodentate G2.5COO(Pt(NH_3_)_2_)_16_ metallodendrimer was characterized with MALDI in the linear mode using the *trans*-2-[3-(4-*tert*-Butylphenyl)-2-methyl-2-propenylidene]malononitrile (DCTB) matrix. The remaining cisplatin-metallodendrimers were characterized by ESI in the positive mode. The metallodendrimers were dissolved in water, and the methanol was used as the ionizing phase. The Elemental analysis (EA) was performed using the Elemental Analysis Unit at Interdepartmental Research Service in Universidad Autónoma de Madrid through the equipment LECO-CHNS 932. FTIR analysis was carried out with a PerkinElmer Spectrum Two spectrometer apparatus. Fluorescence studies were made in a PerkinElmer LS 55 fluorescence spectrometer equipment, and a PerkinElmer UV-Vis spectrometer Lambda was used for the UV-Vis studies. The ζ-potential measurements were performed using a Malvern, Zetasizer Nano ZS equipment with a He–Ne laser and 633 nm wavelength at 25 °C with disposable folded capillary and cuvettes (DTS1060C and ZEN0040, Malvern, respectively). The compounds were dissolved in UPW (ζ-potential determination) and PBS pH 7.4 (size and dispersity (*Đ*), formerly known as polydispersity index (PDI) [35]) with a 0.3 mg/mL concentration and filtered with a 0.2 µm filter before analysis. Five independent experiments were obtained for each sample. 

#### 2.2.1. Synthesis of Cisplatin-Metallodendrimers in a Monodentate Form

##### G0.5COONa PAMAM Dendrimer with Cisplatin–G0.5(COOPt(NH_3_)_2_Cl)_8_

**Scheme 1:**
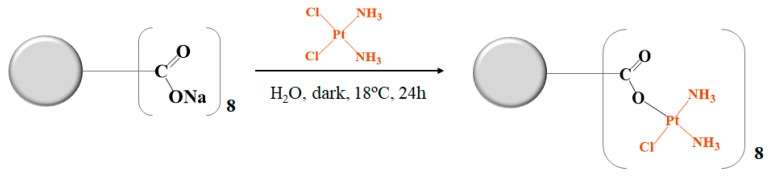
Representation of the synthesis of the PAMAM dendrimer G0.5 functionalized with cisplatin in monodentate form, G0.5(COOPt(NH_3_)_2_Cl)_8_.

Cisplatin (0.12 g, 0.41 mmol; 8.5 equivalent (eq.) mol) was dispersed in 48 mL of UPW. Then, 0.06 g of G0.5COONa PAMAM (0.05 mmol) was dissolved in 3 mL of UPW and added to the mixture. The solution was left for 24 h at 18 °C in the dark under a nitrogen atmosphere with constant mild stirring (Scheme 1). After a while, a change was noticed in the solution, and the cisplatin suspension disappeared, resulting in a clear yellow solution in the end. The solution was then purified with dialysis (MW 100–500 Da) for 4.5 h in distilled water. After freeze-drying, a “greenish-yellow” hygroscopic solid was obtained (0.14 g) with a 91% yield. ^1^H-NMR (400 MHz, D_2_O) [ppm]: δ= 2.63 (H_c_ + H_i_, t, 23H), 2.66 (H_a_, s, 4H), 2.97 (H_g_, t, 8H), 3.26 (H_b_, t, 10H), 3.34 (H_h_, t, 12H), and 3.64 (H_f_, t, 8H). ^13^C-NMR (100 MHz, D_2_O) [ppm]: δ= 30.32 (C_c_ + C_i_), 33.64 (C_a_), 34.25 (C_f_), 48.23 (C_g_), 50.30 (C_h_), 50.99 (C_b_), 174.33 (C_d_), 177.53 (C_p_). ^195^Pt-NMR (86 MHz, D_2_O) [ppm]: δ = −1848.15 (PtO). FTIR (KBr pellet): ν = 1576 cm^−1^ (amide II, N-H and C-N), 1631 cm^−1^ (C=O), 2853 cm^−1^ (C-H), 2966 cm^−1^ (C-H), 3277 cm^−1^ (NH_3_), and 3418 cm^−1^ (N-H). Fluorescence: λ_ex_ = 380 nm, λ_em,_ max = 442 nm. TOF-MS (ESI+) *m*/*z* calc. = 1606.68, *m*/*z* found = 1606.46 [M+2H^+^+H_2_O] C_46_H_118_Cl_8_N_26_O_21_Pt_8_^2+^. EA (%) calc.: C 17.29, H 3.60, N 11.40, found: C 14.96, H 3.92, N 9.20 (hygroscopic compound).

##### G1.5COONa PAMAM Dendrimer with Cisplatin–G1.5(COOPt(NH_3_)_2_Cl)_16_

**Scheme 2:**
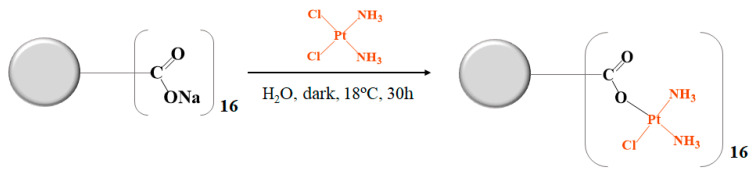
Representation of the synthesis of the PAMAM dendrimer G1.5 functionalized with cisplatin in monodentate form, G1.5(COOPt(NH_3_)_2_Cl)_16_.

Cisplatin (0.29 g, 0.97 mmol; 17.5 eq. mol) was dispersed in 100 mL of UPW. Then, 0.16 g of G1.5COONa PAMAM (0.06 mmol) was dissolved in 8.5 mL of UPW and subsequently added to the cisplatin solution with a constant mild stirring. The solution was left for 30 h, protected from light at 18 °C under a nitrogen atmosphere (Scheme 2). After 5 h, the cisplatin suspension disappeared, obtaining, in the end, a transparent yellow solution. The resulting solution was purified with dialysis (MW 2000 Da) in distilled water for 4.5 h and, after, was freeze-dried. A hygroscopic “greenish-yellow” solid was obtained with a 35% of yield (0.13 g). ^1^H-NMR (400 MHz, D_2_O) [ppm]: δ = 2.65 (H_o_, t, 36H), 2.85 (H_c_ + H_i_, t, 19H), 3.20 (H_a +_ H_g_, m, 14H), 3.30 (H_b_ + H_h_ + Hn, m, 56H), 3.52 (H_m_, t, 18H), and 3.68 (H_f_ + H_l_, m, 23H). ^13^C-NMR (100 MHz, D_2_O) [ppm]: δ= 28.68 (C_c_ + C_i_), 29.80 (C_o_), 33.98 (C_f +_ C_l_), 48.84 (C_a +_ C_g_), 50.22 (C_n_), 51.02 (C_m_), 51.50 (C_h_ + C_b_), 172.36 (C_d_), and 177.16 (C_p_). ^195^Pt-NMR (86 MHz, D_2_O) [ppm]: δ= −1848.02 (PtO). FTIR (KBr pellet): ν = 1587 cm^−1^ (amide II, N-H and C-N), 1658 cm^−1^ (C=O), 2841 cm^−1^ (C-H), 2961 cm^−1^ (C-H), 3251 cm^−1^ (NH_3_) and 3425 cm^−1^ (N-H). Fluorescence: λ_ex_ = 380 nm, λ_em,_ max = 455 nm. TOF-MS (ESI +) *m*/*z* calc. = 1013.47, *m*/*z* found = 1013.46 [M+3H^+^] C_110_H_205_N_30_O_44_Pt_2_^3+^. EA (%) calc.: C 19.43, H 4.03, N 11.95, found: C 38.65, H 7.30, N 11.96 (very hygroscopic compound). 

##### G2.5COONa PAMAM Dendrimer with Cisplatin–G2.5(COOPt(NH_3_)_2_Cl)_32_

**Scheme 3:**
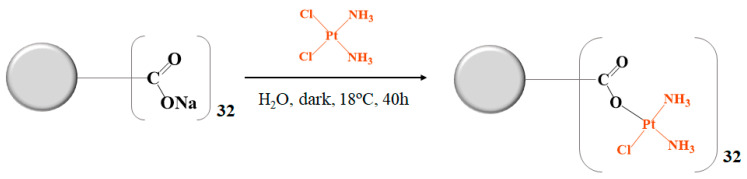
Representation of the synthesis of the PAMAM dendrimer G2.5 functionalized with cisplatin in monodentate form, G2.5(COOPt(NH_3_)_2_Cl)_32_.

Cisplatin (0.094 g, 0.31 mmol; 32 eq. mol) was dispersed in 37 mL of UPW. Under constant mild stirring, an aqueous solution of G2.5COONa PAMAM (0.06 g, 0.01 mmol, 3 mL of UPW) was added to the cisplatin solution. The solution was left for 40 h at 18 °C in the dark under a nitrogen atmosphere (Scheme 3). The cisplatin suspension disappeared after a while, obtaining, in the end, a transparent yellow solution. The solution was purified with dialysis (MW 2000 Da) for 4.5 h in distilled water. A greenish hygroscopic solid was obtained with a 55% yield (0.07 g). ^1^H-NMR (400 MHz, D_2_O) [ppm]: δ= 2.69 (H_c_ + H_i_, H_o_ + H_u_, t, 89H), 2.82 (H_f +_ H_l_, t, 37H), 3.18 (H_t_, t, 50H), 3.45 (H_a_ + H_g_ + H_m_ + H_s_ + H_b_ + H_h_ + H_n_, m, 126H), and 3.74 (H_r_, t, 32H). ^13^C-NMR (100 MHz, D_2_O) [ppm]: δ = 29.46 (C_l_ + C_f_), 29.99 (C_c_ + C_i_ + C_o_ + C_u_), 34.09 (C_r_), 48.83 (C_t_), 50.39 (C_n_), 51.02 (Ca + C_m_ + C_s_), 51.17 (C_h_ + C_h +_ C_n_), 173.22 (C_d_), and 177.20 (C_p_). ^195^Pt-NMR (86 MHz, D_2_O) [ppm]: δ = −1841.86 (PtO). FTIR (KBr pellet): ν = 1581 cm^−1^ (amide II, N-H and C-N), 1647 cm^−1^ (C=O), 2844 cm^−1^ (C-H), 2963 cm^−1^ (C-H), 3263 cm^−1^ (NH_3_), and 3423 cm^−1^ (N-H). Fluorescence: λ_ex_ = 380 nm, λ_em,_ max = 458 nm. TOF-MS (ESI+) *m*/*z* calc. = 974.82, *m*/*z* found = 974.65 [M+H^+^] C_238_H_562_Cl_29_N_116_O_92_Pt_29_^+^. EA (%) calc.: C 20.42, H 4.15, N 12.21, found: C 33.73, H 6.56, N 12.01 (hygroscopic compound). 

#### 2.2.2. Synthesis of Cisplatin-Metallodendrimers in a Bidentate Form

##### Aquation of Cisplatin

**Scheme 4:**
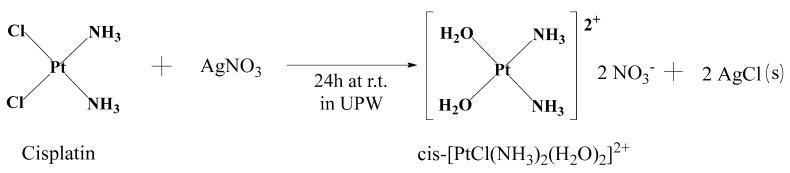
Synthesis of aquated cisplatin.

For the preparation of cisplatin-metallodendrimers in a bidentate form, cisplatin was first hydrolyzed. Accordingly, 0.25 g of cisplatin (0.84 mmol) was dispersed in 100 mL of UPW. Then, an aqueous solution of 0.28 g of AgNO_3_ (1.69 mmol, 2 eq. mol) in 6.5 mL of UPW was added dropwise to the mixture under stirring. The solution was left for 24 h at r.t. in darkness under a nitrogen atmosphere (Scheme 4). After 24 h, a “milky-white” precipitate (silver chloride precipitate) was observed, indicating the formation of the aquated cisplatin. The silver chloride precipitate was removed through centrifugation at 20,124× *g* for 1.5 h at 20 °C. The supernatant was afterward filtrated through a 0.22 µm nylon filter to remove the remaining silver chloride precipitate and freeze-dried. A yellow fluorescent powder was obtained at the end with a 97% yield (0.22 g). 

##### G0.5COONa PAMAM Dendrimer with Cisplatin–G0.5COO(Pt(NH_3_)_2_)_4_

**Scheme 5:**
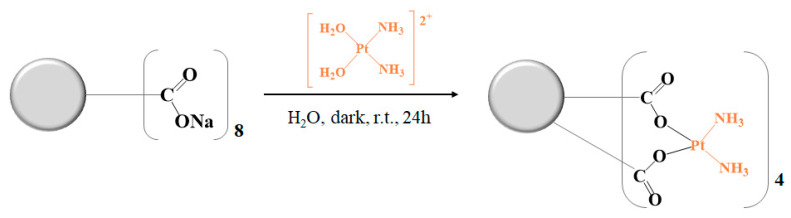
Representation of the synthesis of the PAMAM dendrimer G0.5 functionalized with cisplatin in bidentate form, G0.5COO(Pt(NH_3_)_2_)_4_.

Aquated cisplatin (0.12 mg, 0.42 mmol, 5.5 eq. mol) was dispersed in 46 mL of UPW, and an aqueous solution of 5 mL of G0.5COONa PAMAM (0.10 g, 0.08 mmol) was added to the mixture under stirring. The solution was left for 24 h at r.t. in the dark under a nitrogen atmosphere (Scheme 5). After that time, the resulting solution was purified through dialysis (MW 100–500 Da) in distilled water for 8 h and then freeze-dried, obtaining a green hygroscopic solid with an 80% yield (0.13 g). ^1^H-NMR (400 MHz, D_2_O) [ppm]: δ= 2.57 (H_c_ + H_i_, m, 19H), 2.71 (H_a,_ 3H), 2.92 (H_g_, 9H), 3.19 (H_b_, m, 9H), 3.27 (H_b_, m, 12H), and 3.58 (H_f_, t, 8H). ^13^C-NMR (100 MHz, D_2_O) [ppm]: δ = 31.26 (C_i_), 32.07 (C_c_), 34.96 (C_f_), 48.83 (C_g_), 49.93 (C_a_), 50.58 (C_h_), 51.34 (C_b_), 174.99 (C_d_), and 178.50 (C_p_). ^195^Pt-NMR (86 MHz, D_2_O) [ppm]: δ= −2119.18 (PtO_2_). FTIR (KBr pellet): ν = 1557 cm^−1^ (amide II, N-H and C-N), 1633 cm^−1^ (C=O), 2853 cm^−1^ (C-H), 2923 cm^−1^ (C-H), and 3435 cm^−1^ (N-H). Fluorescence: λ_ex_ = 380 nm, λ_em,_ max = 440 nm. TOF-MS (ESI+) *m*/*z* calc. = 1023.27, *m*/*z* found = 1023.27 [M+2Na^+^] C_46_H_96_N_18_Na_2_O_20_Pt_4_^2+^. EA (%) calc.: C 27.60, H 4.83, N 12.60, found: C 21.93, H 5.71, N 10.60 (hygroscopic compound). 

##### G1.5COONa PAMAM Dendrimer with Cisplatin–G1.5COO(Pt(NH_3_)_2_)_8_

**Scheme 6:**
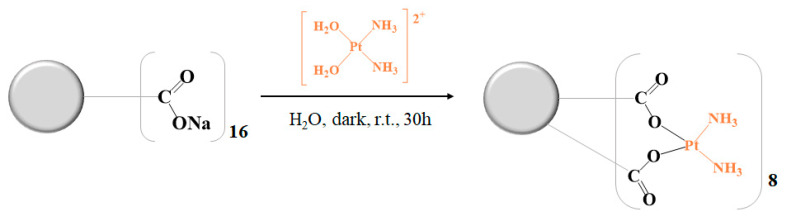
Representation of the synthesis of the PAMAM dendrimer G1.5 functionalized with cisplatin in bidentate form, G1.5COO(Pt(NH_3_)_2_)_8_.

Aquated cisplatin (0.19 g, 0.72 mmol, 8.5 eq. mol) was dispersed in 80 mL of UPW. Therefore, 0.25 g of G1.5COONa PAMAM (0.09 mmol) was dissolved in 10 mL of UPW and added dropwise to the aquated cisplatin mixture under stirring. The mixture was left for 30 h at r.t. in the dark under a nitrogen atmosphere (Scheme 6). The obtained solution was purified through dialysis (MW 2000 Da) for 8 h in distilled water and then freeze-dried. An ivory hygroscopic powder was obtained at the end with a yield of 64% (0.24 g). ^1^H-NMR (400 MHz, D_2_O) [ppm]: δ= 2.53 (H_c_ + H_i_, 30H), 2.61 (H_o,_ 29H), 2.98 (H_a_ + H_g +_ H_m_, 40H), 3.36 (H_n_, H_b_ + H_h_, 56H), and 3.63 (H_f_ + H_l_, 20H). ^13^C-NMR (100 MHz, D_2_O) [ppm]: δ= 30.04 (C_o_), 31.53 (C_c +_ C_i_), 34.07 (C_f +_ C_l_), 48.32 (C_a_ + C_g_), 50.28 (C_n_), 50.95 (C_h +_ C_h_), 52.97 (C_m_), 174.74 (C_d_), and 177.37 (C_p_). ^195^Pt-NMR (86 MHz, D_2_O) [ppm]: δ = −2119.38 (PtO_2_). FTIR (KBr pellet): ν = 1586 cm^−1^ (amide II, N-H and C-N), 1644 cm^−1^ (C=O), 2856 cm^−1^ (C-H), 2976 cm^−1^ (C-H), 3266 cm^−1^ (NH_3_), and 3400 cm^−1^ (N-H). Fluorescence: λ_ex_ = 380 nm, λ_em,_ max = 457 nm. TOF-MS (ESI+) *m*/*z* calc. = 1466.80, *m*/*z* found = 1466.83 [M+3H^+^] C_110_H_227_N_42_O_44_Pt_8_^3+^. EA (%) calc.: C 30.03, H 5.13, N 13.37, found: C 32.43, H 6.34, N 11.72 (hygroscopic compound).

##### G2.5COONa PAMAM Dendrimer with Cisplatin–G2.5COO(Pt(NH_3_)_2_)_16_

**Scheme 7:**
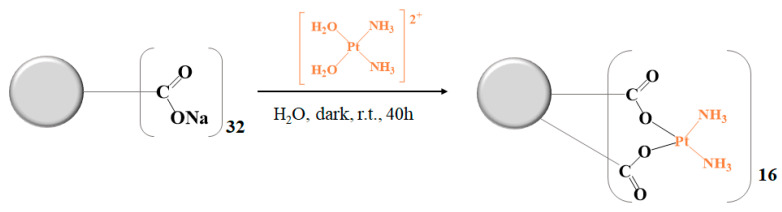
Representation of the synthesis of the PAMAM dendrimer G2.5 functionalized with cisplatin in bidentate form, G2.5COO(Pt(NH_3_)_2_)_16_.

Aquated cisplatin (0.18 g, 0.68 mmol, 16.5 eq. mol) was dispersed in 75 mL of UPW. Separately, 0.26 g of G2.5COONa PAMAM (0.04 mmol) was dissolved in 10 mL of UPW and added dropwise to the aquated cisplatin mixture under stirring. The solution was left for 40 h at r.t. in the dark under a nitrogen atmosphere (Scheme 7). Finally, the solution was purified through dialysis (MW 2000 Da) for 8 h in distilled water and then freeze-dried. A light gray hygroscopic powder was obtained with a yield of 56% (0.21 g). ^1^H-NMR (400 MHz, D_2_O) [ppm]: δ = 2.59 (H_c_ + H_i_ +H_o_, 39H), 2.65 (H_u,_ t_,_ 67H), 3.05 (H_t_, 84H), 3.40 (H_a_ + H_g_ + H_m_, H_s_ + H_b_ + H_h_, H_n_, m, 112H), 3.58 (H_l_ + H_f_, 17H), and 3.68 (H_r_, 32H). ^13^C-NMR (100 MHz, D_2_O) [ppm]: δ= 30.08 (C_c +_ C_i_ + C_o +_ C_u_), 34.13 (C_r_), 36.01 (C_f +_ C_l_), 48.68 (C_t_), 50.41 (C_a_ + C_g_ + C_m_ + C_s_), 51.11 (C_b +_ C_h +_ C_n_), 174.33 (C_d_), and 177.30 (C_p_). ^195^Pt-NMR (86 MHz, D_2_O) [ppm]: δ = −2122.82 (PtO_2_). FTIR (KBr pellet): ν = 1587 cm^−1^ (amide II, N-H and C-N), 1645 cm^−1^ (C=O), 2847 cm^−1^ (C-H), 2966 cm^−1^ (C-H), 3255 cm^−1^ (NH_3_), and 3425 cm^−1^ (N-H). Fluorescence: λ_ex_ = 380 nm, λ_em,_ max = 452 nm. TOF-MS (MALDI) *m*/*z* calc. = 4598.51, *m*/*z* found = 4597.5 [M+2H^+^] C_238_H_482_N_90_O_92_Pt_16_^2+^. EA (%) calc.: C 31.08, H 5.26, N 13.71, found: C 35.63, H 6.98, N 12.56 (hygroscopic compound).

##### G3.5COONa PAMAM Dendrimer with Cisplatin–G3.5COO(Pt(NH_3_)_2_)_32_

**Scheme 8:**
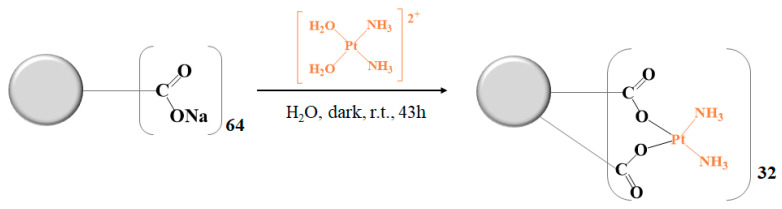
Representation of the synthesis of the PAMAM dendrimer G3.5 functionalized with cisplatin in bidentate form, G3.5COO(Pt(NH_3_)_2_)_32_.

Aquated cisplatin (0.10 g 0.39 mmol, 32.5 eq. mol) was dispersed in 90 mL of UPW. Then, 0.16 g of G3.5COONa PAMAM (0.01 mmol) was dissolved in 10 mL of UPW and added dropwise to the aquated solution under stirring. The solution was left for 43 h at r.t. in darkness under a nitrogen atmosphere (Scheme 8). The mixture was then purified through dialysis (MW 3500 Da) for 1 day in distilled water. After freeze-drying, a light gray hygroscopic powder was obtained with a yield of 58% (0.13 g). ^1^H-NMR (400 MHz, D_2_O) [ppm]: δ= 2.59 (H_c_ + H_i_ +H_o_ + Hu, m 159H), 2.66 (H_Ұ,_ m_,_ 145H), 3.05 (H_a_ + H_g_ + H_m_ + H_s_, 64H), 3.26 (H_y_, 73H), 3.40 (H_z_ + H_b_, H_h_, H_n_, H_t_, m, 253H), 3.59 (H_f_ + H_l_ + H_r_, 36H), and 3.69 (H_x_, 64H). ^13^C-NMR (100 MHz, D_2_O) [ppm]: δ = 29.96 (C_Ұ_), 31.80 (C_c +_ C_i_ + C_o +_ C_u_), 34.07 (C_x_), 35.51 (C_f +_ C_l +_ C_r_), 48.62 (C_a_ + C_g_ + C_m_ + C_s +_ C_y_), 50.37 (C_z_), 51.07 (C_b +_ C_h +_ C_n +_ C_t_), 173.68 (C_d_), and 177.20 (C_p_). ^195^Pt-NMR (86 MHz, D_2_O) [ppm]: δ = −2120.93 (PtO_2_). FTIR (KBr pellet): ν = 1581 cm^−1^ (amide II, N-H and C-N), 1642 cm^−1^ (C=O), 2850 cm^−1^ (C-H), 2927 cm^−1^ (C-H), 2953 cm^−1^ (C-H), and 3466 cm^−1^ (N-H). Fluorescence: λ_ex_ = 380 nm, λ_em,_ max = 454 nm. TOF-MS (ESI +) *m*/*z* calc. = 974.82, *m*/*z* found = 974.64 [M+H^+^] C_494_H_981_N_180_O_188_Pt_29_^+^. EA (%) calc.: C 31.58, H 5.32, N 13.87, found: C 35.01, H 6.87, N 12.89 (hygroscopic compound).

### 2.3. Cell Culture and Cytotoxicity Assays

Six different human cell lines were used to assess the cytotoxicity, namely A2780 (ovarian cancer cell line), A2780cisR (cisplatin-resistant ovarian cancer cell line), CACO-2 (colorectal adenocarcinoma cancer cell line), MCF-7 (breast cancer cell line), and BJ (non-cancer cell line) cells. The A2780 (ECACC 93112519), A2780cisR (ECACC 93112517), and CACO-2 (ECACC 86010202) cells were obtained at the European Collection of Authenticated Cell Cultures (ECACC) service, while the DSMZ-German Collection of Micro-organisms and Cell Cultures GmbH, Leibniz Institute, provided the MCF-7 cell line. The BJ-hTERT (human fibroblasts) cell line was originally from Dr. William C. Hahn Lab. (Dana-Farber Cancer Institute/Harvard Cancer Center, Harvard Medical School, Boston, MA, USA) and generously provided by Dr. Annica K.B. Gad (Department of Oncology and Metabolism, Medical School, University of Sheffield, UK). Cultures were conducted at 37°C, in a humidified atmosphere of 5% CO_2_, in 96-well plates (seeding density = 1 × 10^4^ cells/well), using adequate culture media complemented with fetal bovine serum (FBS) 10% (*v*/*v*) and antibiotic-antimycotic (AA, 100x solution) 1% (*v*/*v*). A2780 and A2780cisR cells were grown in RPMI 1640 medium with L-glutamine (2 mM). Previous to the cyto-toxicity assays, the A2780cisR cells medium also had 1% (*w*/*v*) of cisplatin (100 mM) to guarantee the resistance of cells to that drug. CACO-2 cells were cultured in MEM medium and added to 1% (v/v) nonessential amino acids (NEAA, 100× solution). MCF-7 cells were grown in RPMI 1640 medium with 1mM sodium pyruvate, 1% (*v*/*v*) NEAA, and 3.3 µg/mL human insulin. BJ cells were cultured in D-MEM medium. One day after, cells were incubated with metallodendrimer solutions prepared using nuclease-free water. For a volume total of 200 µL in each well, there was used 100 µL of the sample solution. The following compound concentrations were used in the assays: 0.01, 0.03, 0.1, 0.5, 1, 2.5, 5, and 10 µM. Pristine anionic PAMAM dendrimers (G0.5-G3.5) and free cisplatin were used for control experiments. After 72 h of incubation with the samples, the culture medium was replaced with a new culture medium containing 10% (v/v) of a 0.5 mg/mL solution of MTT (3-[4, 5-dimethylthiazol-2-yl]-2, 5-diphenyltetrazolium bromide). After 2–3 h incubation, the culture medium was removed. The formed formazan crystals were dissolved with 100 µL of DMSO. Then, absorbance was measured at 550 nm using a Victor^3^ 1420 PerkinElmer microplate reader. All the results were obtained from 3 independent experiments with 3 replicas each. IC_50_ values were achieved using linear interpolation between the two experimental points closer to the point corresponding to 50% of the control’s cellular metabolic activity. The results are presented as the mean ± SD (standard deviation).

### 2.4. Hemotoxicity Evaluation

Hemotoxicity studies were performed using red blood cells with the cyanmethemoglobin method [36]. Human blood was collected from healthy donors in EDTA (anticoagulant) containing tubes. For total hemoglobin concentration in human blood assessment, a 250-fold dilution of blood was prepared in C reagent (in an amber bottle, cyanmethemoglobin (C) reagent was prepared by adding 50 mg potassium ferricyanide, 12.5 mg potassium cyanide, and 35 mg potassium dihydrogen phosphate to 250 mL of distilled water containing 250 µL Triton-X; pH was adjusted to 7.4). Then, a bovine hemoglobin (Hg) standard curve was prepared (Figure S1). For that purpose, a stock solution of Hg (1.5 mg/mL) in C reagent was made, and several concentration standards were prepared (0.2; 0.37; 0.54; 0.71; 0.88; 1.05; 1.22, and 1.39 mg/mL). After, absorbance was measured (550 nm) with the C reagent acting as a blank. This standard curve was used to determine total Hg concentration. For metallodendrimers’ hemotoxicity assessment, 10% (*v*/*v*) of a blood solution was prepared in PBS (Mg^2+^/Ca^2+^ free). Then, 10 µL of blood solution was added to a microtube, followed by 70 µL of compound solution (concentrations of 0.1, 1, and 5 µM). Positive and negative controls corresponded to 70 µL of water or PBS, respectively. After incubation at 37 °C for 3 h and centrifugation (1292 ×*g*) for 10 min, 40 µL of each supernatant were transferred to the 96-well plates, and 160 µL of reagent C were added to the distinct wells. Absorbance was measured (550 nm) in the microplate reader Victor^3^ 1420 PerkinElmer. The Hg concentration in the supernatants was calculated using the standard curve. The results are shown as a percentage of hemolysis and were generated from 3 independent assays (mean ± standard deviation (SD)).

### 2.5. DNA Binding Studies by UV-Vis Spectroscopy

Stock solutions of CT-DNA were prepared in 5 mM of Tris-HCl and 50 mM of NaCl (pH 7.4). The DNA purity was previously confirmed with UV-Vis spectroscopy (absorbance at 260 nm/absorbance at 280 nm = 1.9). Absorption spectra were registered at room temperature, at concentrations of 0, 6.25, 12.5, 18.75, 25, 31.25, 37.5, 43.75 to 50 µM in CT-DNA and a constant metallocompound concentration (monodentate G0.5(COOPt(NH_3_)_2_Cl)_8_ (5 µM), G2.5(COOPt(NH_3_)_2_Cl)_32_ (9 µM), bidentate G2.5COO(Pt(NH_3_)_2_)16 (1.5 µM), or cisplatin (9 µM)). The compound solutions started to be prepared in UPW and were then diluted with pH 7.4 buffer (the same buffer used in CT-DNA solutions) to obtain the desired concentration. The solutions containing the different compounds and DNA were incubated for 5 min (room temperature), and the absorbance was measured using a PerkinElmer UV-Vis spectrometer Lambda equipment (the buffer was used as blank). Two independent experiments were performed for each experimental situation. The compound/DNA intrinsic binding constant (K_b_) was calculated using the Benesi–Hildebrand equation [37]. This constant was determined using the ratio of the *y*-intercept to the slope in the plots A_0/_A-A_0_ vs. 1/[DNA]. The ΔG (Gibbs free energy) associated with the DNA interaction process was determined by the equation ∆G = −RTlnK_b_, where R is the gas constant, and T (in Kelvin) is the temperature.

## 3. Results and Discussion

### 3.1. Synthesis and Characterization of Cisplatin-Metallodendrimers

#### 3.1.1. Preparation and Characterization of the Monodentate Form

The preparation of the monodentate cisplatin-metallodendrimers involved the conjugation of cisplatin directly to the anionic half-generation PAMAM dendrimers (G0.5–G2.5). In general, the prepared metallodendrimers were obtained with good to average yields (91% for G0.5(COOPt(NH_3_)_2_Cl)_8_, 35% for G1.5(COOPt(NH_3_)_2_Cl)_16_, and 55% for G2.5(COOPt(NH_3_)_2_Cl)_32_). All the monodentate cisplatin-metallodendrimers were then fully characterized with different techniques such as NMR (^1^H, ^13^C, and ^1^⁹⁵Pt-NMR), FTIR, UV-Vis, fluorescence spectroscopy, ζ-potential, mass spectrometry (MS), and elemental analysis (EA). In the ¹H-NMR experiments, the signal of deuterated water (D_2_O) was used as an internal reference, while potassium tetrachloroplatinate (II) was used as the external reference in the ¹⁹⁵Pt-NMR experiments. Figure S2 shows the ¹H-NMR spectrum of the monodentate G0.5(COOPt(NH_3_)_2_Cl)_8_ with the characteristic signals of the anionic PAMAM dendrimers at 2.65, 2.83, 2.97, 3.26, 3.34, and 3.64 ppm. Compared to the respective pristine PAMAM dendrimer (Figure S3), a downfield shift of the signals was observed, with the protons of the NH_3_ group of cisplatin being obscured by the signal from the deuterated solvent used.

In the ¹³C-NMR spectrum (Figure S4), a downfield shift of the carboxylate signal after the conjugation of cisplatin is observed from 174.69 ppm (Figure S5) to 177.53 ppm, suggesting that the coordination of the PAMAM dendrimer to cisplatin was successfully achieved [34,38]. 

Furthermore, the characterization with ^195^Pt-NMR was performed in order to verify how cisplatin was conjugated to the PAMAM dendrimer. As shown in Figure S6, the signal at −1848 ppm suggests the conjugation of the PAMAM dendrimer with cisplatin in a monodentate form [39]. The signal at −2164 ppm was characteristically attributed to free cisplatin (Figure S7), and the signal at −1756 ppm probably corresponded to *cis*-[PtCl(NH_3_)_2_(D_2_O)]^+^ due to the interaction between the deuterated solvent (D_2_O) and free cisplatin since long periods of acquisition were necessary to obtain the spectrum [40]. The low-intensity signal obtained in the ¹⁹⁵Pt-NMR experiment was due to the sensitivity of the NMR probe used for the ¹⁹⁵Pt-nucleus. Similar results were observed for the G1.5(COOPt(NH_3_)_2_Cl)_16_ and G2.5(COOPt(NH_3_)_2_Cl)_32_ monodentate metallodendrimers (Figures S8–S19). In addition, these monodentate cisplatin-metallodendrimers were evaluated with TOF-MS (ESI positive mode or MALDI) analysis (see the spectra in Figures S20–S22 and Table S1). Although the monodentate cisplatin-metallodendrimers were hygroscopic, it was possible to identify several fragments and/or the expected structure of the prepared metallodendrimers. Prepared monodentate cisplatin-metallodendrimers were also characterized with FTIR. The characteristic bands of cisplatin were present, namely, those corresponding to the N-H bend at 1541 cm^−1^ and the N-H stretch of the amino groups at 3267 cm^−1^ (Figure S23). When comparing with pristine anionic PAMAM dendrimers (Figure S24), cisplatin-metallodendrimers showed a shift in the characteristic carbonyl stretching band from 1639 to 1631 cm^−^¹ in the G0.5(COOPt(NH_3_)_2_Cl)_8_, 1633 to 1658 cm^−1^ in the G1.5(COOPt(NH_3_)_2_Cl)_16_, and 1638 to 1647 cm^−^¹ in the G2.5(COOPt(NH_3_)_2_Cl)_32_ (Figure S25). In addition, the presence of a new band related to the N-H stretch of the NH_3_ groups of cisplatin was observed at 3277 cm^−1^, 3251 cm^−1^, and 3263 cm^−1^ in the G0.5(COOPt(NH_3_)_2_Cl)_8_, G1.5(COOPt(NH_3_)_2_Cl)_16_, and G2.5(COOPt(NH_3_)_2_Cl)_32_ spectra, respectively. The monodentate cisplatin-metallodendrimers were also characterized with UV-Vis spectroscopy (Figure S26). Cisplatin UV-Vis spectrum presented a maximum absorbance band at 203 nm attributed to a charge-transfer band and a shoulder at 280 nm assigned to the d-d transitions of the square planar Pt^2+^ ion [41] (Figure S27a). After the conjugation of cisplatin, the characteristic band around 280–300 nm from the interior tertiary amines of the PAMAM dendrimer (Figure S28) did not significantly shift, suggesting that cisplatin conjugation has been achieved only at the surface of the dendrimer. However, the cisplatin band in the metallodendrimers spectra (a shoulder is visible in the spectra) showed shifts to 205 nm, 250 nm, and 249 nm for G0.5(COOPt(NH_3_)_2_Cl)_8_, G1.5(COOPt(NH_3_)_2_Cl)_16_, and G2.5(COOPt(NH_3_)_2_Cl)_32_, respectively, revealing that the conjugation was successfully achieved. Regarding fluorescence emission, cisplatin has no fluorescence, contrary to the anionic PAMAM dendrimers that present intrinsic fluorescence properties (Figure S27b and S29, respectively). After the coordination of PAMAM dendrimer to cisplatin, it was observed that the fluorescence of G0.5(COOPt(NH_3_)_2_Cl)_8_ slightly increased, and that of the G2.5(COOPt(NH_3_)_2_Cl)_32_ decreased, suggesting the conjugation of cisplatin on the dendrimer surface. However, in the case of G1.5(COOPt(NH_3_)_2_Cl)_16_, the fluorescence increased, making the fluorescence significantly higher than that shown by the other two metallodendrimers (Figure S26b). It appears that fluorescence reached a maximum after the conjugation of the 16 metal complexes, decreasing in the next generation (G2.5(COOPt(NH_3_)_2_Cl)_32_). The explanation for this sudden increase in fluorescence for generation 1.5 (G1.5(COOPt(NH_3_)_2_Cl)_16_) and decrease in generation 2.5 ((G2.5(COOPt(NH_3_)_2_Cl)_32_) is unclear, but it could be related to aggregation and quenching phenomena occurring in the solution.

The ζ-potential analyses obtained in UPW show that, after the coordination of the anionic PAMAM dendrimers to cisplatin, the charge of the prepared cisplatin-metallodendrimers increased as expected. These results suggest that functionalization occurred since cisplatin has a positive charge, and these dendrimers have a negative charge (Table 1). Moreover, it is important to note that the low hemotoxicity observed in the monodentate metallodendrimers (as discussed in Section 3.2.2) is most likely due to their negative ζ values, which affect the surface charge properties and, thus, determine the hemotoxicity of these compounds [42] without affecting their cytotoxicity (see Section 3.2.1). This is the case even though, with the increase of generation, the metallodendrimers become less and less anionic and more neutral. The hydrodynamic size and the dispersity of the pristine dendrimers and metallodendrimers determined in PBS (pH = 7.4) were also analyzed. As expected, the size distribution increased when the dendrimers were functionalized with cisplatin. Their sizes ranged between 173.7 nm (G0.5(COOPt(NH_3_)_2_Cl)_8_), 135.0 nm (G1.5(COOPt(NH_3_)_2_Cl)_16_, and 152.1 nm (G2.5(COOPt(NH_3_)_2_Cl)_32_, following the same trend—a size decrease when the generation increases—as previously reported [32]. The dispersity studies revealed two distinct distribution types for pristine dendrimers and metallodendrimers with cisplatin conjugated in a monodentate form. While pristine dendrimers showed, in general, a high dispersity, with higher generations showing values close to 1, metallodendrimers showed moderate dispersity (ca. 0.3) regardless of generation, and these were very close to the accepted dispersity values for drug delivery applications [43,44].

#### 3.1.2. Preparation and Characterization of the Bidentate Form

The aquation of cisplatin is an important step to assure the removal of the two chlorines and the dendrimer coordination to the metal in a bidentate form. So, the bidentate cisplatin-metallodendrimers were prepared, starting with the aquation of cisplatin using silver nitrate as the two-chlorine abstractor, with a 97% yield. Once the conjugation of the anionic PAMAM dendrimers to cisplatin in a bidentate form involves two bonds, a delay is expected in metallodrug release before the biological target is reached, potentially decreasing the commonly observed side effects. All the synthesized bidentate metallodendrimers were obtained with good yields, namely, 80% for G0.5COO(Pt(NH_3_)_2_)_4_, 64% for G1.5COO(Pt(NH_3_)_2_)_8_, 56% for G2.5COO(Pt(NH_3_)_2_)_16_, and 58% for the G3.5COO(Pt(NH_3_)_2_)_32_. They were fully characterized with NMR (¹H, ¹³C, and ¹⁹⁵Pt-NMR), FTIR, UV-Vis, fluorescence spectroscopy, ζ-potential, MS, and EA. As mentioned before, for the ¹H-NMR experiments, the deuterated water (D_2_O) signal was used as an internal reference, while potassium tetrachloroplatinate (II) was used as an external reference for the ¹⁹⁵Pt-NMR experiments. Figure S30 shows the ¹H-NMR spectrum of the G0.5COO(Pt(NH_3_)_2_)_4_ with the characteristic signals of PAMAM dendrimer between 2.57 to 3.58 ppm. In general, the metallodendrimer signals suffered a downfield shift compared to the pristine PAMAM dendrimer (Figure S3). 

In the ¹³C-NMR spectrum (Figure S31), a downfield shift of the carboxylate group signal from 174.69 to 178.50 ppm was visible, confirming cisplatin conjugation to the PAMAM dendrimer.

The signal at −2119 ppm in the ¹⁹⁵Pt-NMR spectrum (Figure S32) suggests the bidentate conjugation of the anionic PAMAM dendrimer to cisplatin, while the signal at −1545 ppm probably corresponds to *cis*-[PtCl(NH_3_)_2_(H_2_O)_2_]^2+^. Similar results were obtained for the remaining prepared metallodendrimers (Figure S33–S41). Additionally, the conjugation of the anionic PAMAM dendrimers to cisplatin was evaluated with TOF-MS (ESI positive mode or MALDI) analysis (see the spectra in Figures S42–S45 and Table S2). Furthermore, although the bidentate cisplatin-metallodendrimers were hygroscopic, it was possible to identify several fragments and/or the expected structure of the prepared bidentate cisplatin-metallodendrimers. The prepared compounds were also characterized with FTIR (Figure S46). A shift of the carbonyl stretch band was observed when compared to the respective pristine anionic PAMAM dendrimer in all the cisplatin-metallodendrimers spectra (G0.5COO(Pt(NH_3_)_2_)_4_, G1.5COO(Pt(NH_3_)_2_)_8_, G2.5COO(Pt(NH_3_)_2_)_16_, and G3.5COO(Pt(NH_3_)_2_)_32_). In the G0.5COO(Pt(NH_3_)_2_)_4_ spectrum the shift was observed from 1639 cm^−1^ to 1633 cm^−1^, in the G1.5COO(Pt(NH_3_)_2_)_8_ spectrum from 1633 cm^−1^ to 1644 cm^−1^, in the G2.5COO(Pt(NH_3_)_2_)_16_ spectrum from 1638 cm^−1^ to 1645 cm^−1^, and in the G3.5COO(Pt(NH_3_)_2_)_32_ spectrum from 1639 cm^−1^ to 1642 cm^-^¹. The slight shifts indicate that the effect of cisplatin was insignificant due to its neutral behavior [45]. A new band related to the N-H stretch of the NH_3_ groups of cisplatin was visible for the G1.5(COOPt(NH_3_)_2_)_8_ and G2.5(COOPt(NH_3_)_2_)_16_ metallodendrimers at 3266 cm^−1^ and 3255 cm^−1^, respectively. For the remaining metallodendrimers, although a new band was not clearly observable, the existent band was significantly shifted, from 3418 cm^−1^ to 3435 cm^−1^ for the G0.5COO(Pt(NH_3_)_2_)_4_ and from 3421 cm^−1^ to 3446 cm^−1^ for the G3.5COO(Pt(NH_3_)_2_)_32_. This observation can probably be explained by the overlapping of the new band and the N-H band of the amide group of PAMAM dendrimers. The cisplatin-metallodendrimers in a bidentate form were also characterized using UV-Vis and fluorescence spectroscopies (Figure S47). After the coordination of the PAMAM dendrimer to cisplatin in a bidentate form, the cisplatin band shifted from 203 to 247 nm, 242 nm, and 241 nm for G1.5COO(Pt(NH_3_)_2_)_8_, G2.5COO(Pt(NH_3_)_2_)_16_, and G3.5COO(Pt(NH_3_)_2_)_32_, respectively. In the spectra, a shoulder was detected around 260 nm (Figure S47a), which was more evident for the higher generations of the PAMAM dendrimers. However, in the G0.5COO(Pt(NH_3_)_2_)_4_ spectrum, that shoulder was not visible, probably because it has only four metal complexes. In addition, the characteristic absorption band of the anionic PAMAM dendrimers, which appeared around 280–300 nm and was attributed to the interior tertiary amines, did not shift significantly, suggesting that cisplatin has been conjugated only at the dendrimer surface. Since cisplatin has no fluorescence (Figure S27b) and the fluorescence spectra (Figure S47b) show that all the cisplatin-metallodendrimers increased their fluorescence after the coordination of the dendrimer to cisplatin, here cisplatin should be conjugated on the PAMAM dendrimer surface. Nevertheless, the fluorescence intensity increased from G0.5COO(Pt(NH_3_)_2_)_4_ until G2.5COO(Pt(NH_3_)_2_)_16_ and then decreased for G3.5COO(Pt(NH_3_)_2_)_3_. The same behavior was also observed for the monodentate-coordinated dendrimers, with the monodentate G1.5COO(Pt(NH_3_)_2_Cl)_16_ metallodendrimer presenting the highest fluorescence values. Once these two types of metallodendrimers, in mono- and bidentate forms, have 16 metal complexes conjugated on their surface, this seems to indicate that, independently of the conjugation form (mono- or bidentate), the maximum fluorescence in these systems is reached with 16 metals complexes (and does not always increase with the generation, that is, with the presence of more metal complexes).

Furthermore, and as noted above for the monodentate metallodendrimers, the increase in the anionic PAMAM dendrimers **ζ**-potential after the conjugation with cisplatin (Table 2) indicates that the dendrimer’s coordination to cisplatin occurred at the dendrimer surface. Similarly, the variation of the ζ-potential in this family of dendrimers tends toward more positive values with increasing the generation. This behavior is more striking than the one observed for the monodentate metallodendrimers but still supports the observed low hemotoxicity of these dendrimers’ family (see Section 3.2.2). The size distribution of the bidentate metallodendrimers regarding the pristine dendrimers increased when the dendrimers were conjugated with cisplatin. The hydrodynamic sizes ranged between 69.7 to 132.4 nm. However, the difference in size between dendrimer generations is not particularly noticeable. This phenomenon, in the case of pristine dendrimers, at first sight, may be related to the dendrimers’ ability, with the growing generation, to internalize some terminal branches. However, the possibility that the interaction between phosphate ions of the PBS solution and the dendrimers may have contributed to the slight size variations with increasing dendrimer generations cannot be disregarded [46]. As also already observed by us in the monodentate dendrimers and by others [32], in the case of the bidentate metallodendrimers, although the amines groups from conjugated cisplatin could bond hydrogen from water increasing the hydrodynamic size, the interaction with phosphates ions also needs to be considered when analyzing the modest variations in the size of the metallodendrimers with increasing generations. The dispersity of the metallodendrimers is relatively low (0.2–0.4), a typical characteristic of a moderately disperse distribution and similar to that observed for the monodentate metallodendrimers. However, the dispersity values observed for the pristine dendrimers were higher, indicating self-aggregation, a common characteristic of these compounds [47]. 

### 3.2. Biological Studies with the Cisplatin-Metallodendrimers

#### 3.2.1. In Vitro Cytotoxicity Evaluation

Previous studies have indicated that cisplatin is likely to be released from the dendrimer scaffold within cells due to the high concentration of chloride ions in this environment [48,49,50]. The chloride ions will then substitute the dendrimer carboxylate groups in the coordination sphere of platinum (exchange reaction), and the subsequent in situ aquation of cisplatin will give rise to potent electrophilic cations that bind to DNA and cause cytotoxic effects. As such, the cytotoxicity behavior of the cisplatin metallodendrimers in mono- and bidentate forms (G0.5(COOPt(NH_3_)_2_Cl)_8,_ G1.5(COOPt(NH_3_)_2_Cl)_16,_ G2.5(COOPt(NH_3_)_2_Cl)_32_ G0.5(COOPt(NH_3_)_2_)_4_, G1.5(COOPt(NH_3_)_2_)_8_, G2.5(COOPt(NH_3_)_2_)_16_, and G3.5(COOPt(NH_3_)_2_)_32_) was studied in vitro using four cancer cell lines (the A2780, A2780cisR, MCF-7, and CACO-2 cell lines) and one non-cancer cell line (the BJ cell line). The MTT assay that measures the metabolic activity of cells was used for cytotoxicity evaluation after 72 h of cell culture. The results are presented in Table 3 as the half-maximal inhibitory concentration (IC_50_) values and can be analyzed regarding the cell line, the dendrimer generation, and the type of coordination to cisplatin. 

The obtained IC_50_ values showed that all the metallodendrimers exhibit cytotoxicity against the tested cancer cell lines within the range of studied concentrations and that the monodentate metallodendrimers G0.5(COOPt(NH_3_)_2_Cl)_8_ and G2.5(COOPt((NH_3_)_2_Cl)_32_ were globally the most cytotoxic (considering all cancer cell lines). The CACO-2 cells proved to be mostly resistant to most of the compounds tested, including cisplatin.

Different parameters were determined to compare the synthesized metallodendrimers with cisplatin regarding their efficacy, namely the relative potency, selectivity, and resistance factor. The cytotoxicity relative potency (RP) value was determined by dividing the IC_50_ of cisplatin by the IC_50_ of the metallodendrimer (Table 4). The results revealed that the metallodendrimers are particularly toxic against cisplatin-resistant cells (represented by the A2780cisR cancer cell line), for which cisplatin presents clinical limitations [51]. For this cell line, the monodentate G0.5(COOPt(NH_3_)_2_Cl)_8_ metallodendrimer presented a RP value of 400, followed by the G2.5(COOPt((NH_3_)_2_Cl)_32_ metallodendrimer with a RP value of 100 (the bidentate G3.5(COOPt((NH_3_)_2_)_32_, and the G2.5(COOPt((NH_3_)_2_)_16_ metallodendrimers showed RP values of 67 and 24, respectively. Interestingly, a trend in cytotoxicity was not observed with the increase in dendrimer generation (increased number of metallic centers). However, to some extent, the type of dendrimer coordination to cisplatin seems to have a role in the anti-cancer effectiveness of the two metallodendrimers, with the highest RP values corresponding to a monodentate form. Possibly, cisplatin is more easily released as it establishes only one bond with the dendrimer scaffold. Indeed, on average, the RP values for the monodentate systems are higher than for the bidentate ones. 

Another limitation of platinum drugs is the selectivity for cancer cells, leading to undesirable side effects. In this sense, the cytotoxicity of the metallodendrimers was also determined for the non-cancer BJ cell line to evaluate the cancer selectivity of the synthesized metallodendrimers. The selectivity index (SI) of the metallodendrimers shown in Table 5 is defined as the ratio between the IC_50_ obtained for normal cells (BJ cells in the present case) and the IC_50_ obtained for cancer cells. According to the literature, when the SI is greater than 2, the compound has good selectivity for cancer cells [51,52,53]. If the SI values are less or equal to 2, the compound only presents general toxicity. 

Cisplatin itself and all the metallodendrimers had SI values greater than 2 when considering the A2780 cancer cell line, indicating that the compounds were more cytotoxic to this cancer cell line than the non-cancer cell line. In this regard, four metallodendrimers showed enhanced selectivity compared with cisplatin, the monodentate G2.5(COOPt((NH_3_)_2_Cl)_32_, and the bidentateG0.5(COOPt(NH_3_)_2_)_4_, G1.5(COOPt(NH_3_)_2_)_8_, and G3.5(COOPt((NH_3_)_2_)_32_ metallodendrimers. In the case of A2780cisR cancer cells, all the metallodendrimers presented higher SI values than cisplatin, except the G1.5(COOPt(NH_3_)_2_Cl)_16_ and G1.5COO(Pt(NH_3_)_2_)_8_ metallodendrimers (four of them are selective for these cells). The compounds with the highest SI values were the monodentate G0.5(COOPt(NH_3_)_2_Cl)_8_ (SI value < 6) and G2.5(COOPt(NH_3_)_2_Cl)_32_ (SI value = 10) metallodendrimers and the bidentate G2.5COO(Pt(NH_3_)_2_)_16_ (SI value = 3) and G3.5COO(Pt(NH_3_)_2_)_32_ (SI value = 20) metallodendrimers. These results indicate that they can be considered promising anti-cancer drugs with a SI value several times higher than cisplatin. On the other hand, in the case of MCF-7 and CACO-2 cell lines, all SI values were inferior to 2 (also for cisplatin), only evidencing general toxicity.

However, it is important to emphasize that for a compound to be ideally selective, the selectivity index, that is, the ratio between its cytotoxicity and its biological activity must be ≥ 10. If the SI is low (<1), the compound is toxic and, therefore, cannot be used as a safe drug [54,55,56].

Since the beginning of the development of new platinum anti-cancer drugs, researchers have aimed at overcoming resistance to cisplatin. The A2780 and A2780cisR cancer cells were used to determine the resistance factor (RF) of the metallodendrimers. The RF is defined as the ratio between the IC_50_ value for A2780cisR cells and the IC_50_ value for A2780 cells. The lower the RF value, the better the compound overcomes cancer cells’ resistance to the drug [32]. As can be seen in Table 6, except for the bidentate G1.5(COOPt(NH_3_)_2_)_8_ metallodendrimer, all other metallodendrimers showed RF values significantly lower than cisplatin (RF = 36), and the monodentate G1.5(COOPt((NH_3_)_2_Cl)_16_ is nearly similar to cisplatin. The metallodendrimers with an RF lower than 1 will be more effective in overcoming the drug resistance of cancer cells. The metallodendrimers that presented lower RF were the following: the monodentate G0.5(COOPt(NH_3_)_2_Cl)_8_ with RF value < 0.5, followed by the monodentate G2.5(COOPt((NH_3_)_2_Cl)_32_ with RF value = 0.6, and the bidentate G2.5(COOPt((NH_3_)_2_)_16_ with RF value = 0.9. These metallodendrimers present attractive advantages once they can help surpass cells’ cisplatin resistance. It should also be highlighted that the use of dendrimers as drug carriers, as is the focus of the present work, may help in vivo accumulation of the drug in the tumor by enhancing the permeability and retention (EPR) effect, which may occur in some instances [57].

#### 3.2.2. Hemotoxicity Assay

Hemotoxicity assay was used to study the interaction of the prepared cisplatin-metallodendrimers with red blood cells by measuring the release of hemoglobin through the membrane disruption of these cells. Following this and based on the results (Figure S48), all the metallodendrimers and cisplatin showed very low hematoxicity levels, less than 5% for all the concentrations, compared with the values of hemoglobin release of the negative control. The low hematoxicity presented by the metallodendrimers was less or quite similar when compared to cisplatin. However, at 5 µM concentration, cisplatin displays hematoxicity values of 1.2- to 1.5-fold higher than the metallodendrimers. This observation agrees with what is found in the literature, that the ability of cisplatin to induce hematological toxicity is concentration-dependent [8]. According to Mei-Hua Han et al. [58], anionic PAMAM dendrimers have a very low generation and concentration-dependent effect on hemolysis compared to cationic PAMAM dendrimers. Furthermore, these results suggest that the number of platinum metal centers and the generation of the anionic PAMAM dendrimers did not interfere with the toxicity in red blood cells at the concentrations used in the experiment since no significant differences were observed in the hematoxicity. 

#### 3.2.3. DNA Binding Studies

In many chemotherapy approaches, DNA is the main target of the medicines. For instance, cisplatin binds covalently to the DNA through intra- and inter-strand crosslinks with the nitrogens in the DNA bases [37]. This binding mode is irreversible, causing the inhibition of DNA processes and leading to cell death [37]. Cisplatin binds to DNA first through monodentate adducts and then through bidentate adducts forming crosslinks with DNA [59,60,61]. This monoadduct has a long lifetime and plays a vital role in the cytotoxicity of the drug, where the type of the formed crosslink is crucial [60]. As the mechanisms of action of the present metallodendrimers can go beyond cisplatin delivery and subsequent cisplatin interaction with cellular DNA and metallodendrimer/DNA interaction, studies were also investigated in this work.

**Figure 1:**
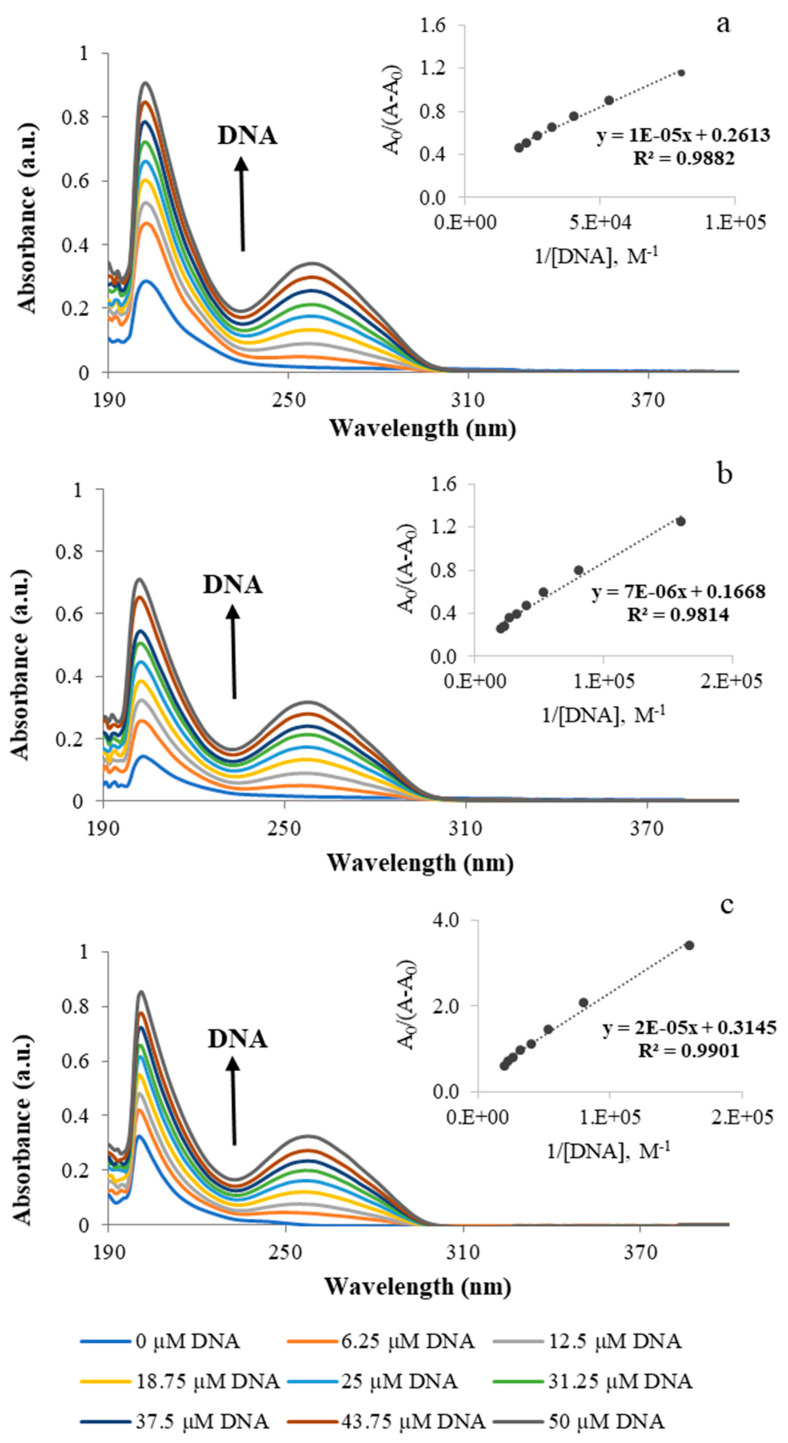
Representative UV-visible spectra of (**a**) monodentate G0.5COO(Pt(NH_3_)_2_Cl)_8_, (**b**) monodentate G2.5COO(Pt(NH_3_)_2_Cl)_32_, and (**c**) bidentate G2.5COO(Pt(NH_3_)_2_)_16_ with increasing concentration of CT-DNA (0, 6.25, 12.5, 18.75, 25, 31.25, 37.5, 43.75, and 50 µM) in 5 mM Tris-HCl/50 mM NaCl at pH 7.4. The inset corresponds to the plot of A_0_/(A − A_0_) versus 1/[DNA], used to determine the binding constant. The arrow indicates the direction of increasing the concentration of DNA.

The interactions of selected metallodendrimers (monodentate G0.5(COOPt(NH_3_)_2_Cl)_8_, G2.5(COOPt(NH_3_)_2_Cl)_32_, and bidentate G2.5COO(Pt(NH_3_)_2_)_16_) with calf thymus DNA (CT-DNA) were studied in vitro using UV-Vis spectroscopy. Cisplatin was also studied for comparison. Figure 1 shows the absorbance spectra of solutions containing different CT-DNA concentrations and a constant concentration of cisplatin or metallodendrimers. The CT-DNA spectra displayed a broad band at 260 nm due to the cytosine, adenine, thymine, and guanine chromophore groups. The spectra of the three metallodendrimers in the presence of increasing CT-DNA concentrations presented a hyperchromic effect. This effect was also noticed for cisplatin (Figure S49), which is known to form covalent adducts with DNA, distorting its conformation [62]. Indeed, the hyperchromic effect caused by cisplatin happens from the presence of charged cations, which readily react with DNA through crosslinks, where two bases of DNA are linked together with the drug. However, for the metallodendrimers, a full release of cisplatin during the 5 min incubation period is not expected to occur, especially for the metallodendrimers with bidentate coordination, even in the presence of chloride ions. In fact, one may expect to have in solution the free cisplatin, fully functionalized dendrimers, and dendrimers that have already partially lost the metallic fragment. The metallodendrimers may even interact with DNA through other interactions (different from those used by free cisplatin), including electrostatic ones, leading to conformational changes and DNA helix disruption.

The DNA binding constant (K_b_) of the compounds with CT-DNA was determined with UV-Vis spectroscopy through the Benesi–Hildebrand equation, specifically from the ratio of the *y*-intercept to the slope in the plots A_0_/A − A_0_ vs. 1/[DNA] [37]. The metallodendrimers showed a strong binding affinity with DNA compared to cisplatin (Table 7). However, the monodentate G0.5COO(Pt(NH_3_)_2_Cl)_8_ and G2.5(COOPt(NH_3_)_2_Cl)_32_ metallodendrimers had a K_b_ value higher than the bidentate G2.5COO(Pt(NH_3_)_2_)_16_. Probably, this is due to the bidentate coordination mode and a more difficult release of the metallodrug from the dendrimer scaffold during the incubation time. In summary, the metallodendrimers present high K_b_ values, indicating a strong interaction with the CT-DNA in the in vitro experiments. Moreover, the Gibbs free energy of the binding process was also determined, and the negative values indicated a spontaneous process (Table 7). Nonetheless, it is important to emphasize that the interactions observed in vitro may not be precisely what happens in vivo. 

## 4. Conclusions

In the present work, anionic half-generation PAMAM dendrimers (G0.5-G3.5) were successfully coordinated to cisplatin in two different forms, mono- and bidentate coordination. The prepared metallodendrimers were cytotoxic against a set of cell lines representative of different types of cancer (A2780, A2780cisR, MCF-7, and CACO-2 cells), and the extent of cytotoxicity was dependent on the cell line and the type of coordination. Globally, the monodentate coordination resulted in lower IC_50_ values, in agreement with a more accessible cisplatin release from the dendritic scaffold. Furthermore, the metallodendrimers were generally more cytotoxic (higher relative potency (RP)) than cisplatin, especially for the A2780 and A2780cisR cell lines, for which they showed higher selectivity when compared with non-cancer cells (BJ cells). The monodentate G0.5COO(Pt(NH_3_)_2_Cl)_8_ and G2.5COO(Pt(NH_3_)_2_Cl)_32_ metallodendrimers, together with the bidentate G2.5COO(Pt(NH_3_)_2_)_16_ metallodendrimer, were even more active towards the cisplatin-resistant cell line (A2780cisR cells) than the correspondent cisplatin-sensitive one (A2780 cells). A trend between cytotoxicity and dendrimer generation (number of transported metallic fragments) was not observed. Additionally, erythrocyte hemolysis caused by metallodendrimers was not significant at the tested concentrations. Regarding the interactions between the metallodendrimers and CT-DNA, our study revealed that strong interactions could be established between both entities. In brief, we demonstrated that the cisplatin coordination mode (monodentate or bidentate) to the dendritic scaffold effectively impacts the in vitro biological properties of the studied metallodendrimers more significantly than the increase in the number of metals, with the monodentate form presenting, in some cases, advantages over the bidentate form. 

## Figures and Tables

**Table 1:** ζ-potential, size, and dispersity of anionic PAMAM dendrimers (G0.5-G3.5) and their related metallodendrimers after conjugation with cisplatin in a monodentate form.

Compounds	ζ-Potential (mV) (in UPW)	Size (nm) (in PBS)	Dispersity (in PBS) *
G0.5(COONa)_8_	−19 ± 1	131.0 ± 22.2	0.357 ± 0.1
G0.5(COOPt(NH_3_)_2_Cl)_8_	−20.7 ± 2.1	173.7 ± 45.1	0.335 ± 0.03
G1.5(COONa)_16_	−40.8 ± 0.7	85.6 ± 29.5	1.000 ± 0.0
G1.5(COOPt(NH_3_)_2_Cl)_16_	−4.5 ± 1.9	135.0 ± 25.4	0.307 ± 0.1
G2.5(COONa)_32_	−48 ± 2	89.7 ± 23.3	0.923 ± 0.1
G2.5(COOPt(NH_3_)_2_Cl)_32_	−0.6 ± 0.9	152.1 ± 13.9	0.301 ± 0.04

* Formerly known as polydispersity index (PDI = *Đ*) [35].

**Table 2:** **ζ**-potential, size, and dispersity of anionic PAMAM dendrimers (G0.5-G3.5) and their related metallodendrimers after conjugation with cisplatin in a bidentate form.

Compounds	ζ-Potential (mV) (in UPW)	Size (nm) (in PBS)	Dispersity (in PBS) *
G0.5(COONa)_8_	−19 ± 1	131.0 ± 22.2	0.357 ± 0.1
G0.5COO(Pt(NH_3_)_2_)_4_	−3.2 ± 1.8	69.7 ± 20.1	0.343 ± 0.1
G1.5(COONa)_16_	−40.8 ± 0.7	85.6 ± 29.5	1.000 ± 0.0
G1.5COO(Pt(NH_3_)_2_)_8_	−3.1 ± 0.9	128.6 ± 19.9	0.253 ± 0.03
G2.5(COONa)_32_	−48 ± 2	89.7 ± 23.3	0.923 ± 0.1
G2.5COO(Pt(NH_3_)_2_)_16_	12.4 ± 1.0	132.4 ± 10.5	0.438 ± 0.05
G3.5(COONa)_64_	- 51 ± 1	88.5 ± 25.1	0.940 ± 0.1
G3.5COO(Pt(NH_3_)_2_)_32_	2.2 ± 2.7	128.6 ± 17.3	0.330 ± 0.04

* Formerly known as polydispersity index (PDI = *Đ*) [35].

**Table 3:** IC_50_ values of the prepared metallodendrimers towards various cancer and non-cancer cell lines. Results are expressed as mean ± SD of three independent experiments performed in triplicate.

	A2780 IC_50_ ± SD (µM)	A2780cisR IC_50_ ± SD (µM)	MCF-7 IC_50_ ± SD (µM)	CACO-2 IC_50_ ± SD (µM)	BJ IC_50_ ± SD (µM)
Cisplatin	0.11 ± 0.03	4 ± 1	1.2 ± 0.5	>10	0.6 ± 0.2
Monodentate form					
G0.5(COOPt(NH_3_)_2_Cl)_8_	0.02 ± 0.01	<0.01	0.08 ± 0.01	3 ± 2	0.06 ± 0.01
G1.5(COOPt((NH_3_)_2_Cl)_16_	0.20 ± 0.08	6.6 ± 0.2	0.5 ± 0.2	>10	0.5 ± 0.2
G2.5(COOPt((NH_3_)_2_Cl)_32_	0.07 ± 0.03	0.04 ± 0.03	0.6 ± 0.3	6 ± 3	0.4 ± 0.2
Bidentate form					
G0.5(COOPt(NH_3_)_2_)_4_	0.3 ± 0.1	5 ± 2	2.0 ± 0.2	8 ± 2	3 ± 1
G1.5(COOPt(NH_3_)_2_)_8_	0.07 ± 0.04	6.4 ± 0.2	1.7 ± 0.6	>10	0.5 ± 0.3
G2.5(COOPt((NH_3_)_2_)_16_	0.18 ± 0.03	0.17 ± 0.05	2.4 ± 0.6	>10	0.5 ± 0.3
G3.5(COOPt((NH_3_)_2_)_32_	0.04 ± 0.02	0.06 ± 0.04	3.3 ± 0.7	>10	1.2 ± 0.5

**Table 4:** Relative potency (RP) of the metallodendrimers *.

Relative Potency (RP)	A2780	A2780cisR	MCF-7	CACO-2	BJ
Monodentate form					
G0.5(COOPt(NH_3_)_2_Cl)_8_	5.5	>400	15	>3.3	10
G1.5(COOPt((NH_3_)_2_Cl)_16_	0.6	0.6	2.4	**	1.2
G2.5(COOPt((NH_3_)_2_Cl)_32_	1.6	100	2.0	>1.7	1.5
Bidentate form					
G0.5(COOPt(NH_3_)_2_)_4_	0.4	0.8	0.6	>1.3	0.2
G1.5(COOPt(NH_3_)_2_)_8_	1.6	0.6	0.7	**	1.2
G2.5(COOPt((NH_3_)_2_)_16_	0.6	24	0.5	**	1.2
G3.5(COOPt((NH_3_)_2_)_32_	2.8	67	0.4	**	0.5

* The relative potency (RP) of the metallodendrimers was calculated from the division of the IC_50_ value of cisplatin by the IC_50_ value of metallodendrimers. ** Cannot be determined.

**Table 5:** Selectivity index (SI) for cisplatin and the metallodendrimers *.

Selectivity Index (SI)	A2780	A2780cisR	MCF-7	CACO-2
Cisplatin	5	0.2	0.5	<0.06
Monodentate form				
G0.5(COOPt(NH_3_)_2_Cl)_8_	3	>6	0.8	0.02
G1.5(COOPt((NH_3_)_2_Cl)_16_	2.5	0.08	1.0	<0.05
G2.5(COOPt((NH_3_)_2_Cl)_32_	5.7	10	0.7	0.07
Bidentate form				
G0.5(COOPt(NH_3_)_2_)_4_	10	0.6	1.5	0.4
G1.5(COOPt(NH_3_)_2_)_8_	7.1	0.08	0.3	<0.05
G2.5(COOPt((NH_3_)_2_)_16_	2.8	2.9	0.2	<0.05
G3.5(COOPt((NH_3_)_2_)_32_	30	20	0.4	<0.1

* The selectivity index (SI) for cisplatin and the metallodendrimers was calculated from the division of the IC_50_ value obtained for the BJ cell line by that obtained for each cancer cell line.

**Table 6:** Resistance factor for cisplatin and the metallodendrimers *.

		Resistance Factor (RF)
	Cisplatin	36
Monodentate form	G0.5(COOPt(NH_3_)_2_Cl)_8_	<0.5
	G1.5(COOPt((NH_3_)_2_Cl)_16_	33
	G2.5(COOPt((NH_3_)_2_Cl)_32_	0.6
Bidentate form	G0.5(COOPt(NH_3_)_2_)_4_	17
	G1.5(COOPt(NH_3_)_2_)_8_	91
	G2.5(COOPt((NH_3_)_2_)_16_	0.9
	G3.5(COOPt((NH_3_)_2_)_32_	1.5

* The resistance factor for cisplatin and the metallodendrimers was calculated from the division of the IC_50_ value obtained for A2780cisR cells by the IC_50_ value obtained for A2780 cells.

**Table 7:** Values of DNA binding constant (K_b_) and Gibbs free energy (ΔG) of the monodentate G0.5COO(Pt(NH_3_)_2_Cl)_8_, bidentate G2.5COO(Pt(NH_3_)_2_)_16_, monodentate G2.5COO(Pt(NH_3_)_2_Cl)_32_ metallodendrimers, and free drug cisplatin. Data are represented as mean ± SD of two independent experiments.

	K_b_ × 10^3^	−ΔG/ KJ mol^−1^
Cisplatin	1.3 ± 0.3	17 ± 1
G0.5COO(Pt(NH_3_)_2_Cl)_8_	26.6 ± 0.7	25 ± 1
G2.5COO(Pt(NH_3_)_2_Cl)_32_	20 ± 6	24 ± 1
G2.5COO(Pt(NH_3_)_2_)_16_	14 ± 3	23 ± 1

## Data Availability

The data presented in this study are available on request from the corresponding author.

## Supplementary Materials

The following supporting information can be downloaded at: https://www.mdpi.com/article/10.3390/pharmaceutics15020689/s1.
