# Supplementary material for: Biological Effects in Cancer Cells of Mono- and Bidentate Conjugation of Cisplatin on PAMAM Dendrimers: A Comparative Study
Source: Pharmaceutics. 2023 Feb 17;15(2):689. doi: 10.3390/pharmaceutics15020689 (PMC9960565; doi:10.3390/pharmaceutics15020689)
Supplement: Supplementary file 1 [file pharmaceutics-15-00689-s001.zip › pharmaceutics-2072190-supplementary.pdf]

Supplementary materials

Biological effects in cancer cells of mono and bidentate conjugation of cisplatin on PAMAM dendrimers: a comparative study

Cláudia Camacho[1], Dina Maciel[1], Helena Tomás[1] and João Rodrigues [1*]

[1]CQM - Centro de Química da Madeira, MMRG, Universidade da Madeira, Campus da Penteada, 9000-390 Funchal, Portugal

*Correspondence: joaoc@staff.uma.pt

Figure Index

Figure S1: Standard curve of Hg using several dilutions: 0.2; 0.37; 0.54; 0.71; 0.88; 1.05; 1.22 and 1.39 mg/mL. The absorbance was measured at 550 nm. 5

Figure S2: ^{1}H-NMR spectrum of monodentate G0.5(COOPt(NH$_3$)$_2$Cl)$_8$ in D$_2$O. 5

Figure S3: ^{1}H-NMR spectrum of anionic PAMAM dendrimer G0.5(COONa)$_8$ in D$_2$O. 6

Figure S4: ^{13}C-NMR spectrum of monodentate G0.5(COOPt(NH$_3$)$_2$Cl)$_8$ in D$_2$O. 6

Figure S5: ^{13}C-NMR spectrum of anionic PAMAM dendrimer G0.5(COONa)$_8$ in D$_2$O. 6

Figure S6: ^{195}Pt-NMR spectrum of monodentate G0.5(COOPt(NH$_3$)$_2$Cl)$_8$ in D$_2$O, with K$_2$PtCl$_4$ as external reference (-1631 ppm). 7

Figure S7: ^{195}Pt-NMR spectrum of cisplatin in D$_2$O, with K$_2$PtCl$_4$ as external reference (-1631 ppm). 7

Figure S8: ^{1}H-NMR spectrum of anionic PAMAM dendrimer G1.5(COONa)$_{16}$ in D$_2$O. 7

Figure S9: ^{13}C NMR spectrum of anionic PAMAM dendrimer G1.5(COONa)$_{16}$ in D$_2$O. 8

Figure S10: ^{1}H-NMR spectrum of anionic PAMAM dendrimer G2.5(COONa)$_{32}$ in D$_2$O. 8

Figure S11: ^{13}C-NMR spectrum of anionic PAMAM dendrimer G2.5(COONa)$_{32}$ in D$_2$O. 9

Figure S12: ^{1}H-NMR spectrum of anionic PAMAM dendrimer G3.5(COONa)$_{64}$ in D$_2$O. 9

Figure S13: ^{13}C-NMR spectrum of anionic PAMAM dendrimer G3.5(COONa)$_{64}$ in D$_2$O. 10

Figure S14: ^{1}H-NMR spectrum of monodentate G1.5(COOPt(NH$_3$)$_2$Cl)$_{16}$ in D$_2$O. 10

Figure S15: ^{13}C NMR spectrum of monodentate G1.5(COOPt(NH_3)$_2$Cl)$_{16}$ in D_2O. 11

Figure S16: ^{195}Pt-NMR spectrum of monodentate G1.5(COOPt(NH_3)$_2$Cl)$_{16}$ in D_2O, with K_2PtCl_4 as an external reference (-1631 ppm). 11

Figure S17: ^{1}H-NMR spectrum of monodentate G2.5(COOPt(NH_3)$_2$Cl)$_{32}$in D_2O. 12

Figure S18: ^{13}C-NMR spectrum of monodentate G2.5(COOPt(NH_3)$_2$Cl)$_{32}$ in D_2O. 12

Figure S19: ^{195}Pt-NMR spectrum of monodentate G2.5(COOPt(NH_3)$_2$Cl)$_{32}$ in D_2O, with K_2PtCl_4 as an external reference (-1631 ppm). 13

Figure S20: TOF-MS (ESI+) mass spectrum of monodentate G0.5(COOPt(NH_3)$_2$Cl)$_8$ metallodendrimer. 14

Figure S21: TOF-MS (ESI+) mass spectrum of monodentate G1.5(COOPt(NH_3)$_2$Cl)$_{16}$ metallodendrimer. 14

Figure S22: TOF-MS (ESI+) mass spectrum of monodentate G2.5(COOPt(NH_3)$_2$Cl)$_{32}$ metallodendrimer. 15

Figure S23: FTIR spectra of cisplatin in KBr pellet. 15

Figure S24: FTIR spectra of different generations of anionic PAMAM dendrimers (G0.5-G3.5) in KBr pellet. 16

Figure S25: FTIR spectra of metallodendrimers conjugated with cisplatin in monodentate form. The spectra were performed in KBr pellet 16

Figure S26: a) Absorption spectra of cisplatin-metallodendrimers in the monodentate form recorded at a concentration of 40 μM in ultrapure water and b) Emission (λ_{ex} = 380 nm) spectra of cisplatin-metallodendrimers in the monodentate form recorded at a concentration of 500 μM in ultrapure water. 17

Figure S27: a) Absorption spectra of cisplatin recorded at a concentration of 40 μM of in ultrapure water and b) Emission (λ_{ex} = 380 nm) spectra of cisplatin recorded at a concentration of 500 μM in ultrapure water. 17

Figure S28: UV-Vis spectra of anionic PAMAM dendrimers at a concentration of 500 μM in ultrapure water. 18

Figure S29: Emission (λ_{ex}= 380 nm) of anionic PAMAM dendrimers at a concentration of 500 μM in ultrapure water. 18

Figure S30: ^{1}H-NMR spectrum of bidentate G0.5(COOPt(NH_3)$_2$)$_4$ in D_2O. 19

Figure S31: ^{13}C-NMR spectrum of bidentate G0.5(COOPt(NH_3)$_2$)$_4$ in D_2O. 19

Figure S32: ^{195}Pt-NMR spectrum of bidentate G0.5COO(Pt(NH_3)$_2$)$_4$ in D_2O, with K_2PtCl_4 as external reference (-1631 ppm)........ 20

Figure S33: ^{1}H-NMR spectrum of bidentate G1.5COO(Pt(NH_3)$_2$)$_8$ in D_2O. 20

Figure S34: ^{13}C-NMR spectrum of bidentate G1.5COOPt(NH_3)$_2$)$_8$ in D_2O. 21

Figure S35: ^{195}Pt-NMR spectrum of bidentate G1.5COO(Pt(NH_3)$_2$)$_8$ in D_2O, with K_2PtCl_4 as external reference (-1631 ppm)........ 21

Figure S36: ^{1}H-NMR spectrum of bidentate G2.5COO(Pt(NH_3)$_2$)$_{16}$ in D_2O. 22

Figure S37: ^{13}C-NMR spectrum of bidentate G2.5COO(Pt(NH_3)$_2$)$_{16}$ in D_2O. 22

Figure S38: ^{195}Pt-NMR spectrum of bidentate G2.5COO(Pt(NH_3)$_2$)$_{16}$ in D_2O, with K_2PtCl_4 as external reference (-1631 ppm)........ 23

Figure S39: ^{1}H-NMR spectrum of bidentate G3.5COO(Pt(NH_3)$_2$)$_{32}$ in D_2O........ 23

Figure S40: ^{13}C-NMR spectrum of bidentate G3.5COO(Pt(NH_3)$_2$)$_{32}$ in D_2O........ 24

Figure S41: ^{195}Pt-NMR spectrum of bidentate G3.5COO(Pt(NH_3)$_2$)$_{32}$ in D_2O, with K_2PtCl_4 as external reference (-1631 ppm)........ 24

Figure S42: TOF-MS (ESI+) mass spectrum of bidentate G0.5COO(Pt(NH_3)$_2$)$_4$ metallodendrimer.... 25

Figure S43: TOF-MS (ESI+) mass spectrum of bidentate G1.5COO(Pt(NH_3)$_2$)$_8$ metallodendrimer.... 26

Figure S44: TOF-MS (MALDI) mass spectrum of bidentate G2.5COO(Pt(NH_3)$_2$)$_{16}$ metallodendrimer. 26

Figure S45: TOF-MS (ESI+) mass spectrum of bidentate G3.5COO(Pt(NH_3)$_2$)$_{32}$ metallodendrimer... 27

Figure S46: FTIR spectrum of metallodendrimers conjugated with cisplatin in bidentate form. The spectrum was performed in KBr pellet. 27

Figure S47: a) Absorption spectra of cisplatin-metallodendrimers in the bidentate form recorded at a concentration of 40 μM in ultrapure water and b) Emission (λ_{ex} = 380 nm) spectra of cisplatin-metallodendrimers in the bidentate form recorded at a concentration of 500 μM in ultrapure water. 28

Figure S48: Hematoxicity of the free cisplatin and prepared cisplatin-metallodendrimers in healthy human blood. Blood was treated for 3 h with different concentrations (0.1, 1, and 5 μM) of the metallodendrimers and free cisplatin. The positive and negative control are represented by C$^+$ and C, respectively. The results are expressed as mean ± SD of at least three independent experiments performed in triplicate. 28

Figure S49: Representative UV-visible spectra of cisplatin with increasing concentration of CT-DNA (0, 6.25, 12.5, 18.75, 25, 31.25, 37.5, 43.75 and 50 μM) in 5 mM Tris-HCl/50 mM NaCl at pH 7.4. The

inset corresponds to the plot of $A_0/(A-A_0)$ versus 1/[DNA], which is used to determine the binding constant. The arrow indicates the direction of increasing the concentration of DNA. 29

Table Index

Table S1: Molecular weight of the cisplatin-metallodendrimers in a monodentate form.................. 13

Table S2: Molecular weight of the cisplatin-metallodendrimers in a bidentate form. 25

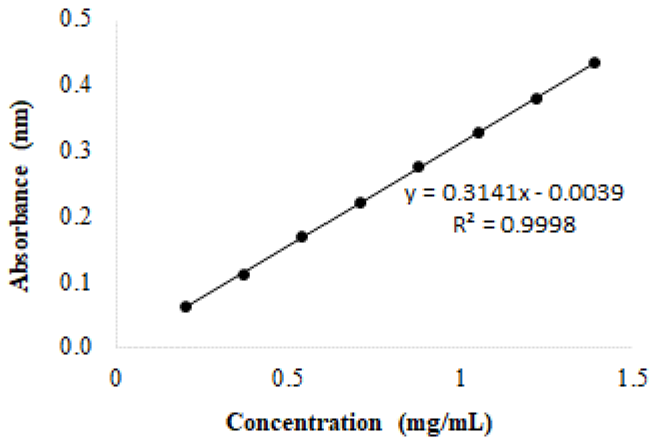

Figure S1: Standard curve of Hg using several dilutions: 0.2; 0.37; 0.54; 0.71; 0.88; 1.05; 1.22 and 1.39 mg/mL. The absorbance was measured at 550 nm.

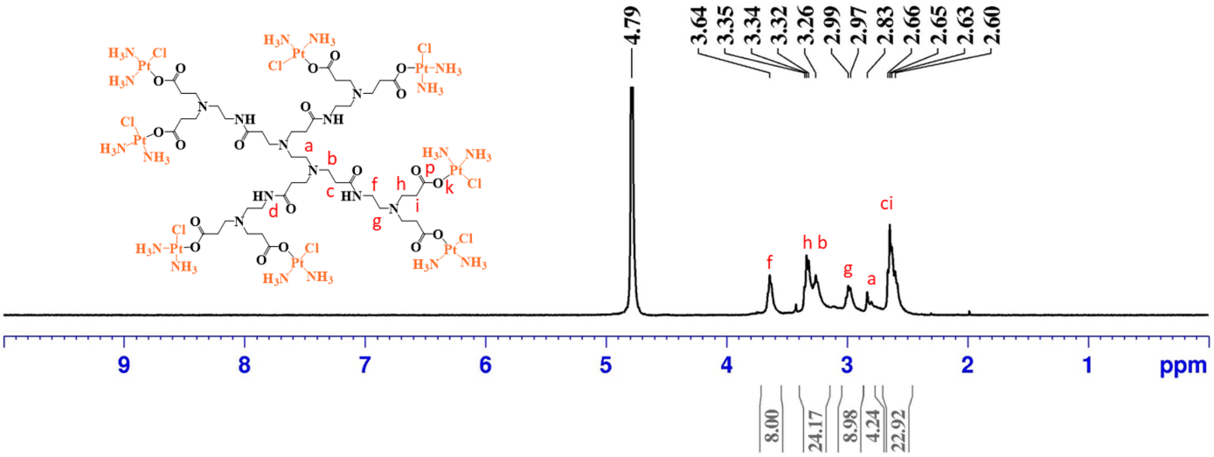

Figure S2: ^{1}H-NMR spectrum of monodentate G0.5(COOPt$(NH_3)_2$Cl$)_8$ in D_2O.

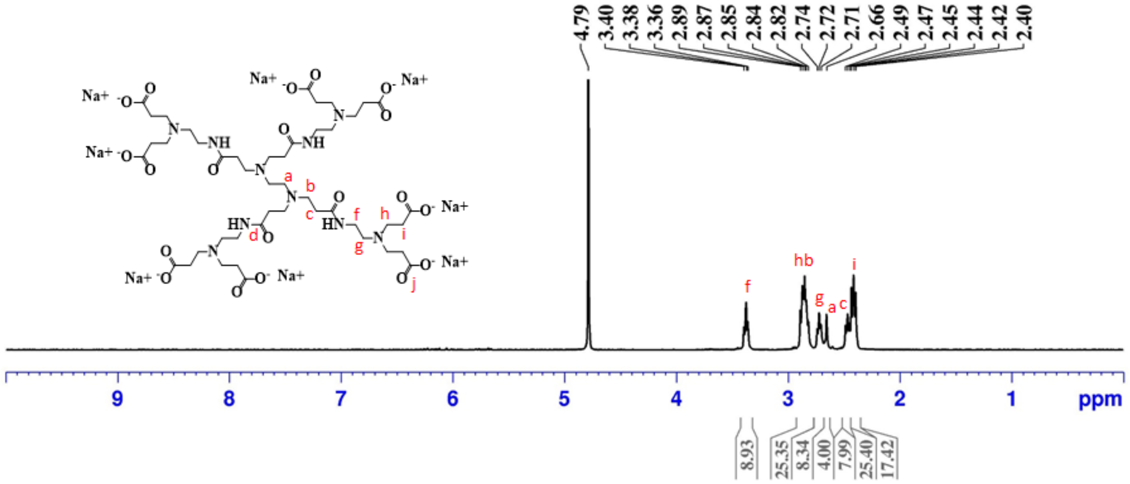

Figure S3: ^{1}H-NMR spectrum of anionic PAMAM dendrimer G0.5(COONa)$_8$ in D_2O.

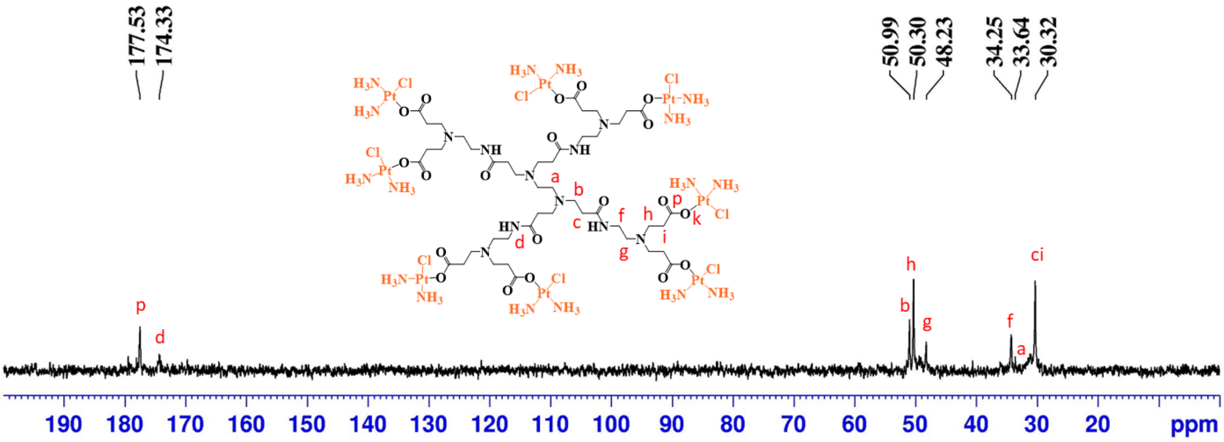

Figure S4: ^{13}C-NMR spectrum of monodentate G0.5(COOPt(NH$_3$)$_2$Cl)$_8$ in D_2O.

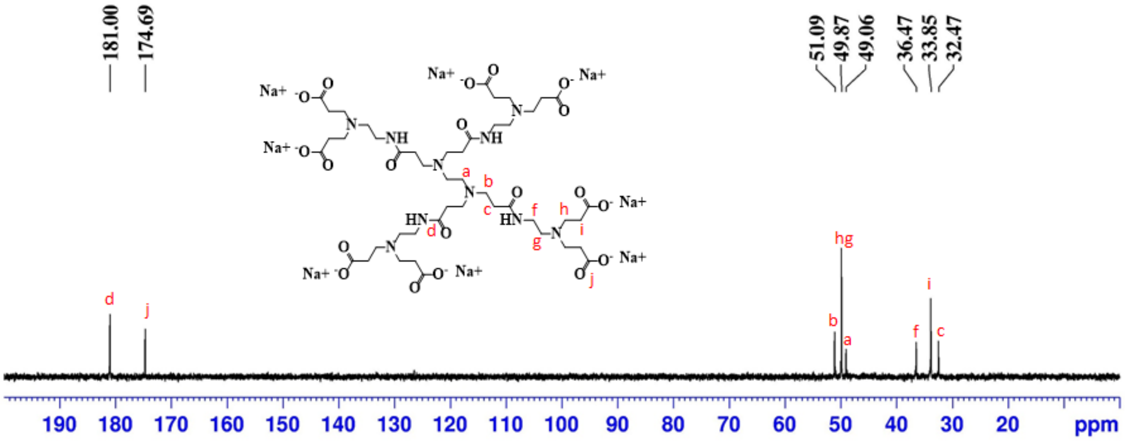

Figure S5: ^{13}C-NMR spectrum of anionic PAMAM dendrimer G0.5(COONa)$_8$ in D_2O.

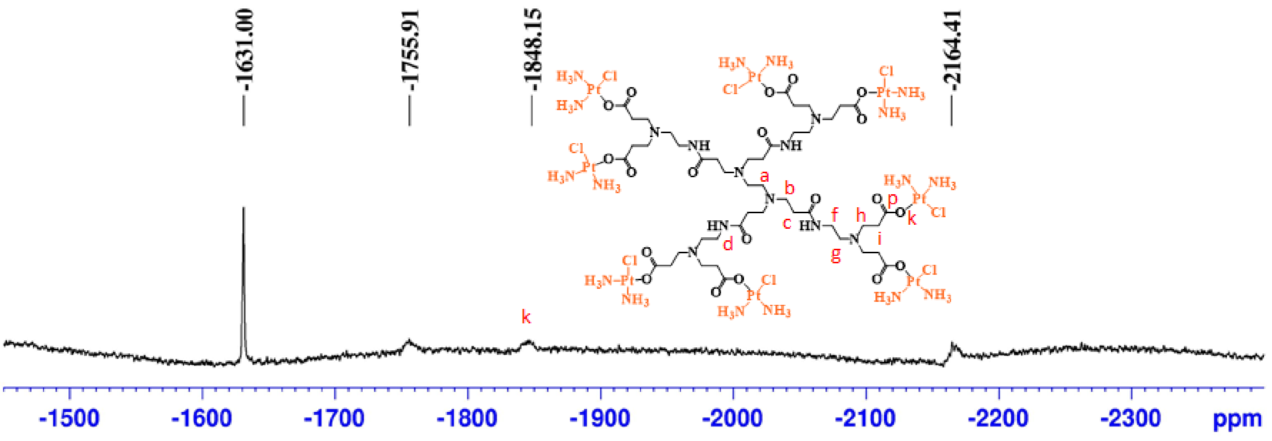

Figure S6: ^{195}Pt-NMR spectrum of monodentate G0.5(COOPt(NH_3)$_2$Cl)$_8$ in D_2O, with K_2PtCl_4 as external reference (-1631 ppm).

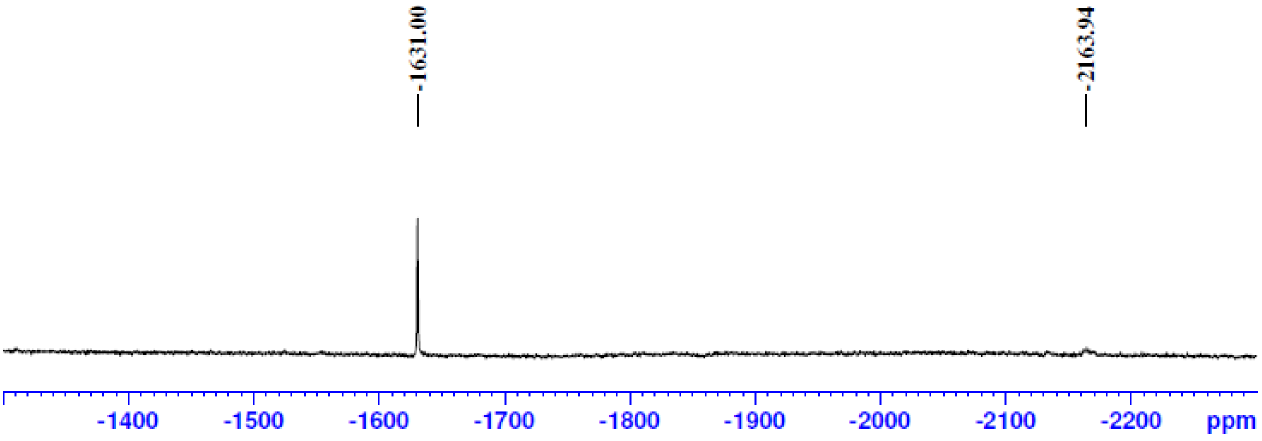

Figure S7: ^{195}Pt-NMR spectrum of cisplatin in D_2O, with K_2PtCl_4 as external reference (-1631 ppm).

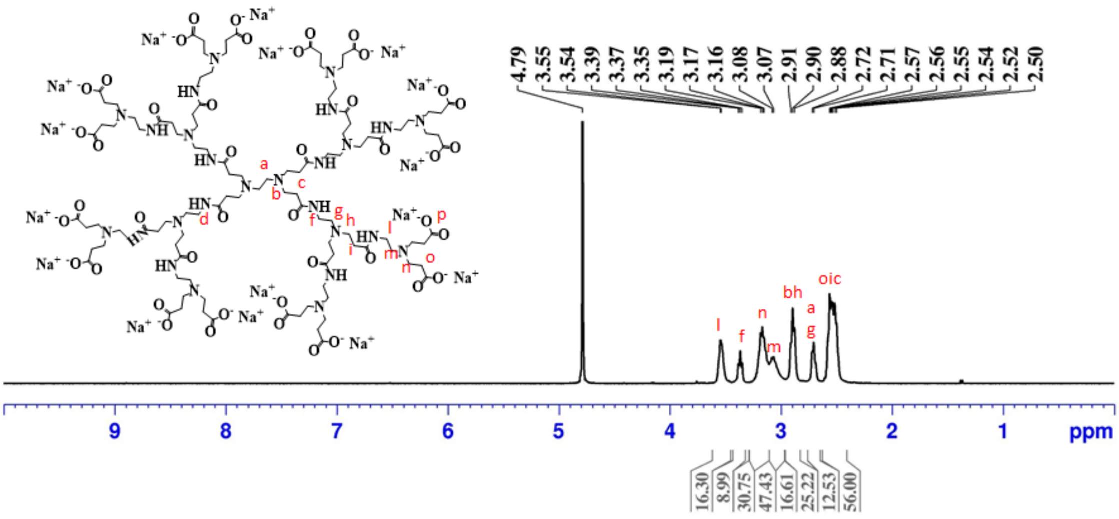

Figure S8: ^{1}H-NMR spectrum of anionic PAMAM dendrimer G1.5(COONa)$_{16}$ in D_2O.

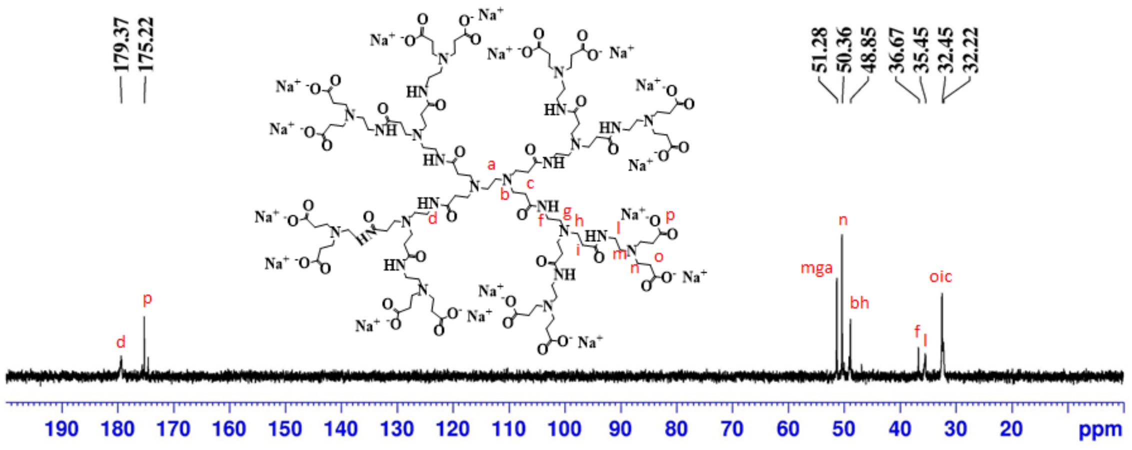

Figure S9: ^{13}C NMR spectrum of anionic PAMAM dendrimer G1.5$(COONa)_{16}$ in D_2O.

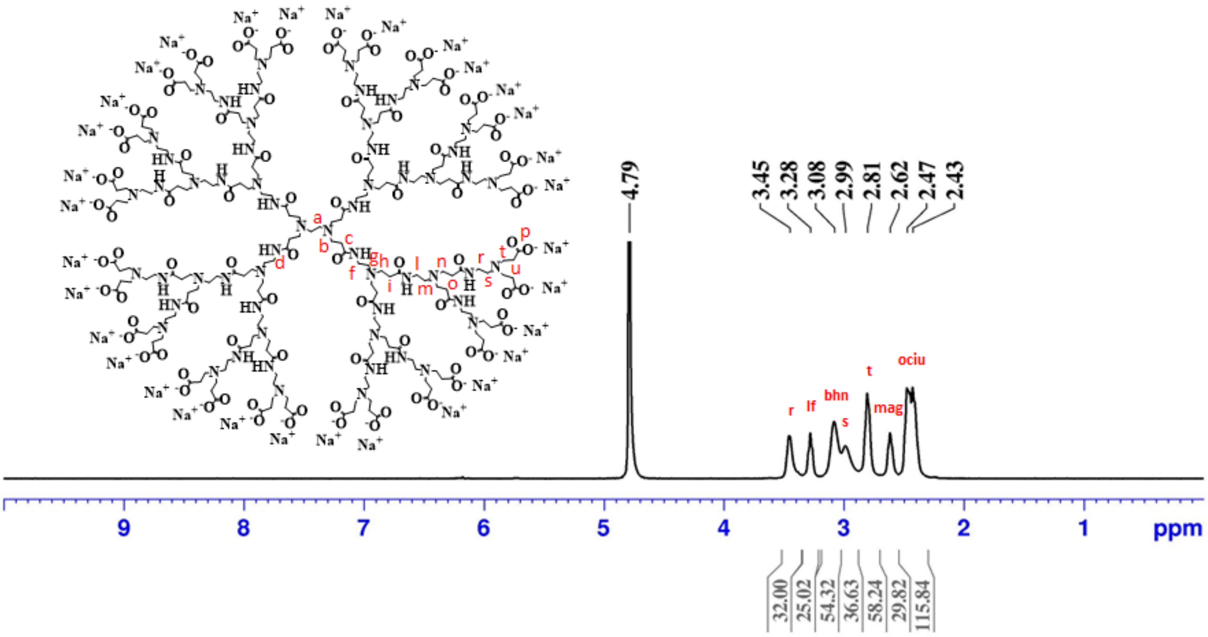

Figure S10: ^{1}H-NMR spectrum of anionic PAMAM dendrimer G2.5$(COONa)_{32}$ in D_2O.

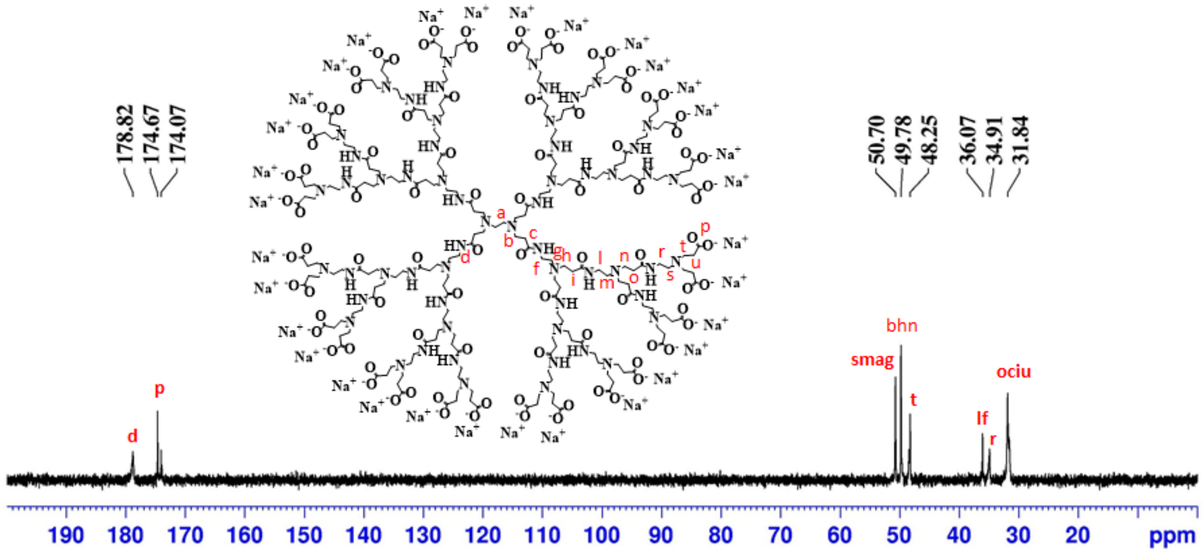

Figure S11: ^{13}C-NMR spectrum of anionic PAMAM dendrimer G2.5(COONa)$_{32}$ in D_2O.

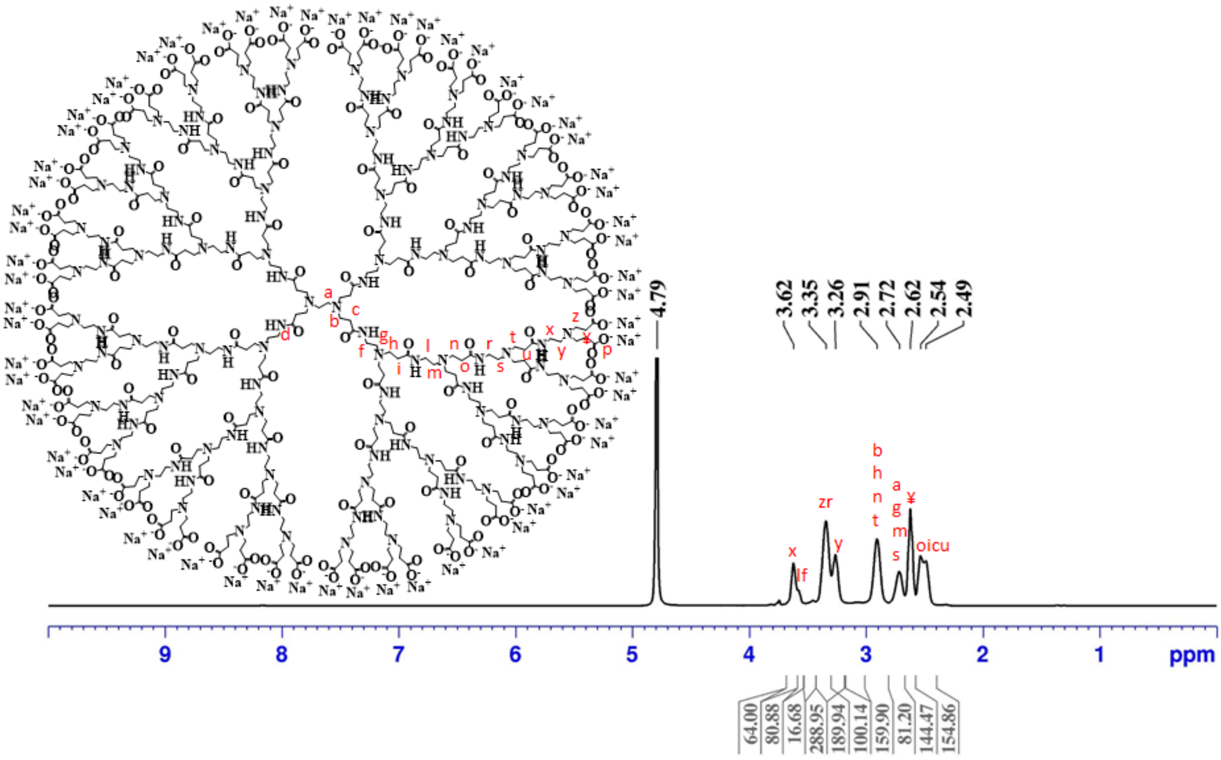

Figure S12: ^{1}H-NMR spectrum of anionic PAMAM dendrimer G3.5(COONa)$_{64}$ in D_2O.

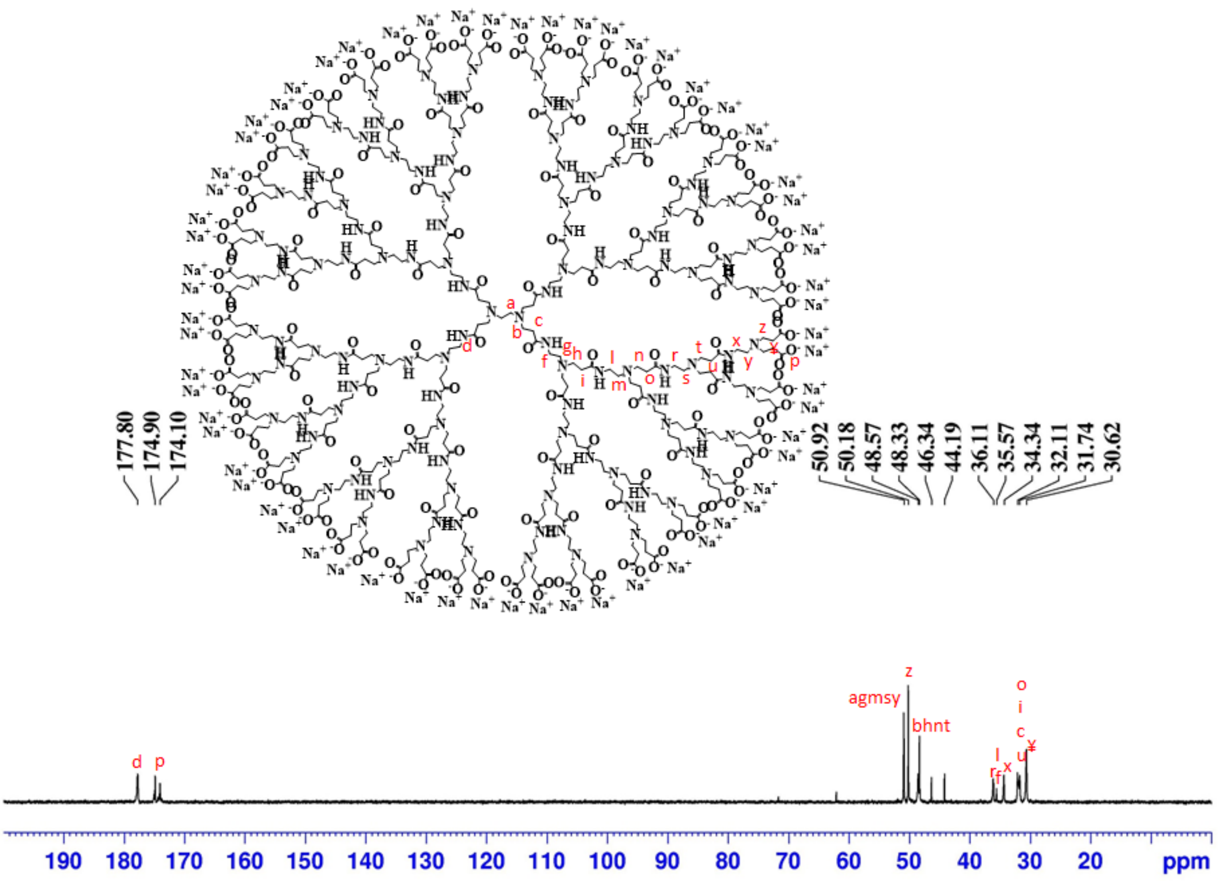

Figure S13: ^{13}C-NMR spectrum of anionic PAMAM dendrimer G3.5$(COONa)_{64}$ in D_2O.

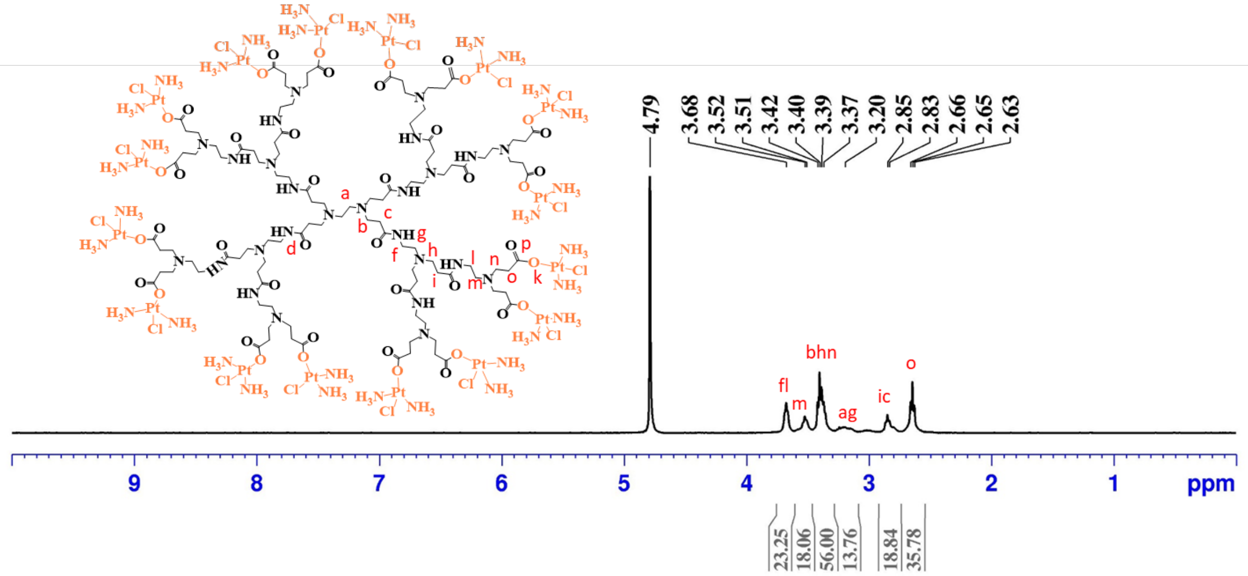

Figure S14: ^{1}H-NMR spectrum of monodentate G1.5$(COOPt(NH_3)_2Cl)_{16}$ in D_2O.

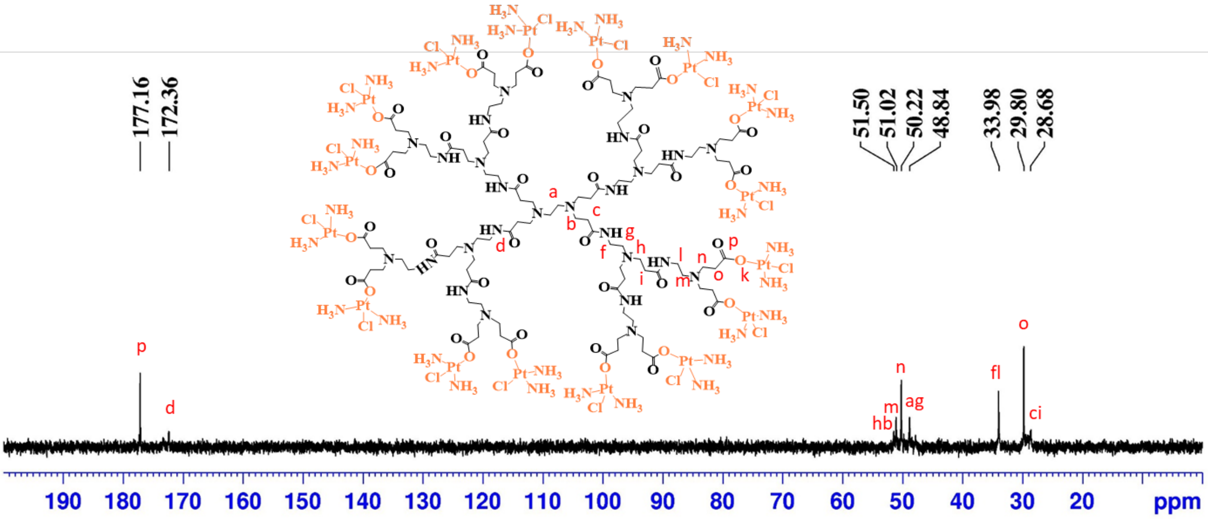

Figure S15: ^{13}C NMR spectrum of monodentate G1.5(COOPt(NH_3)$_2$Cl)$_{16}$ in D_2O.

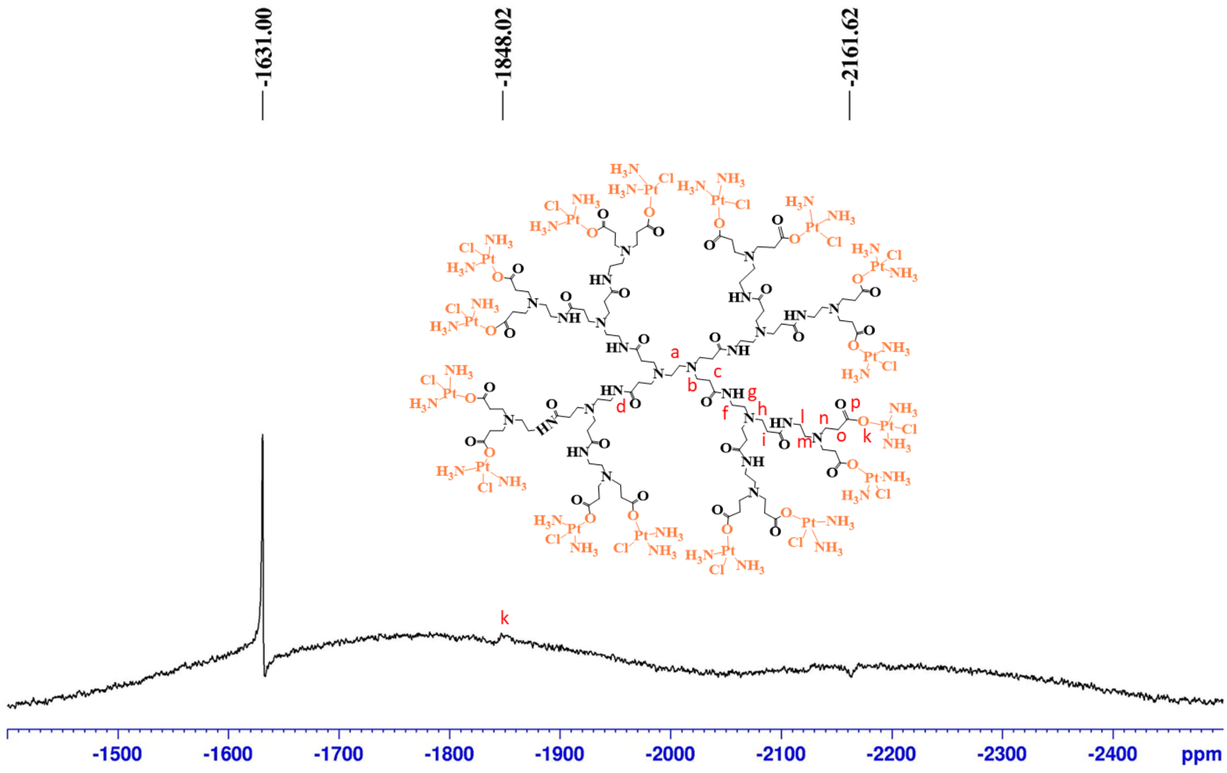

Figure S16: ^{195}Pt-NMR spectrum of monodentate G1.5(COOPt(NH_3)$_2$Cl)$_{16}$ in D_2O, with K_2PtCl_4 as an external reference (-1631 ppm).

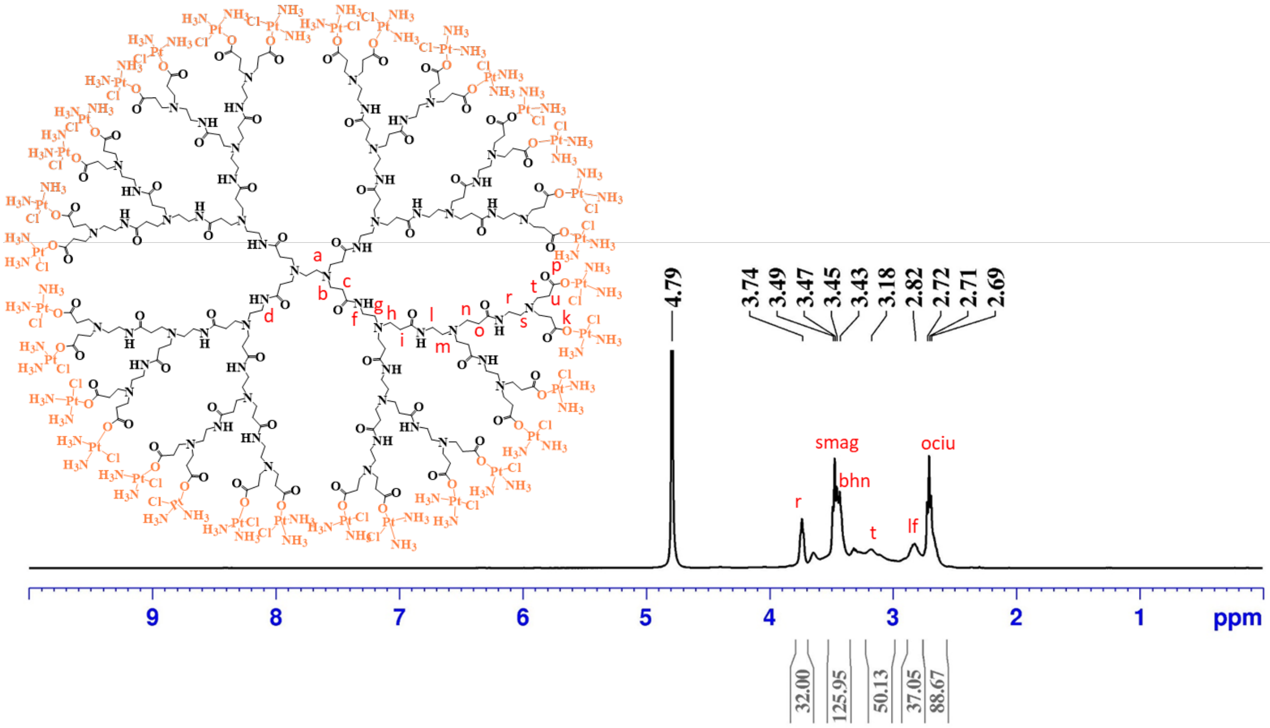

Figure S17: ^{1}H-NMR spectrum of monodentate G2.5(COOPt(NH$_3$)$_2$Cl)$_{32}$in D$_2$O.

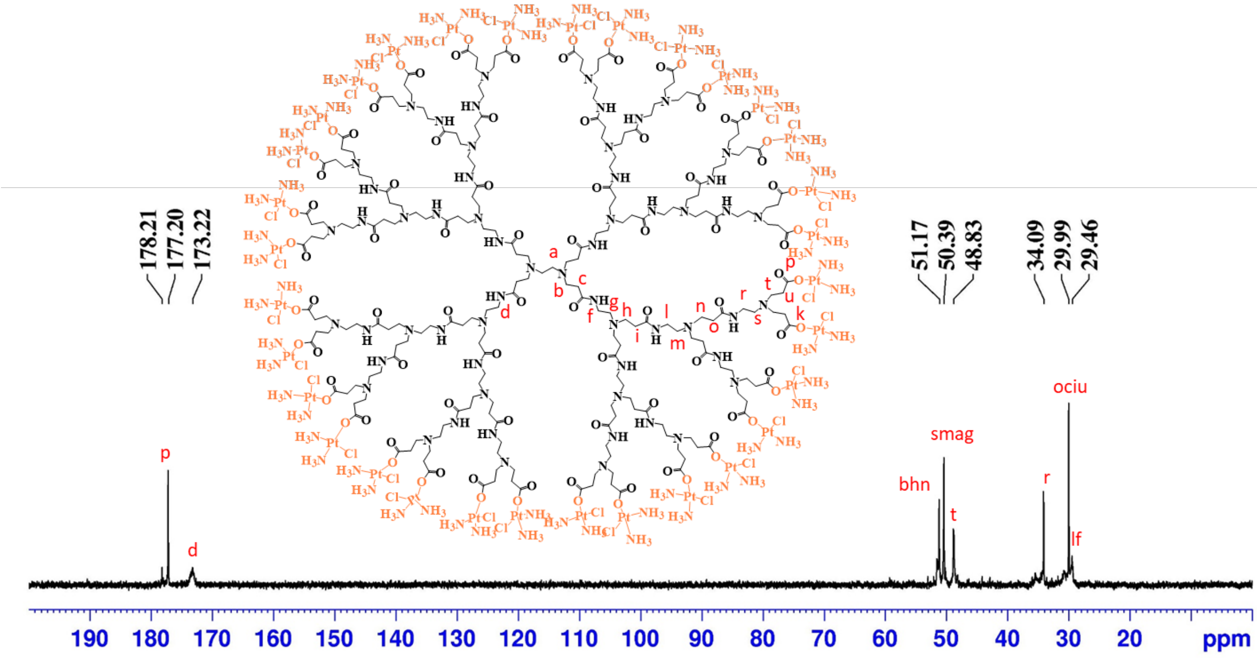

Figure S18: ^{13}C-NMR spectrum of monodentate G2.5(COOPt(NH$_3$)$_2$Cl)$_{32}$ in D$_2$O.

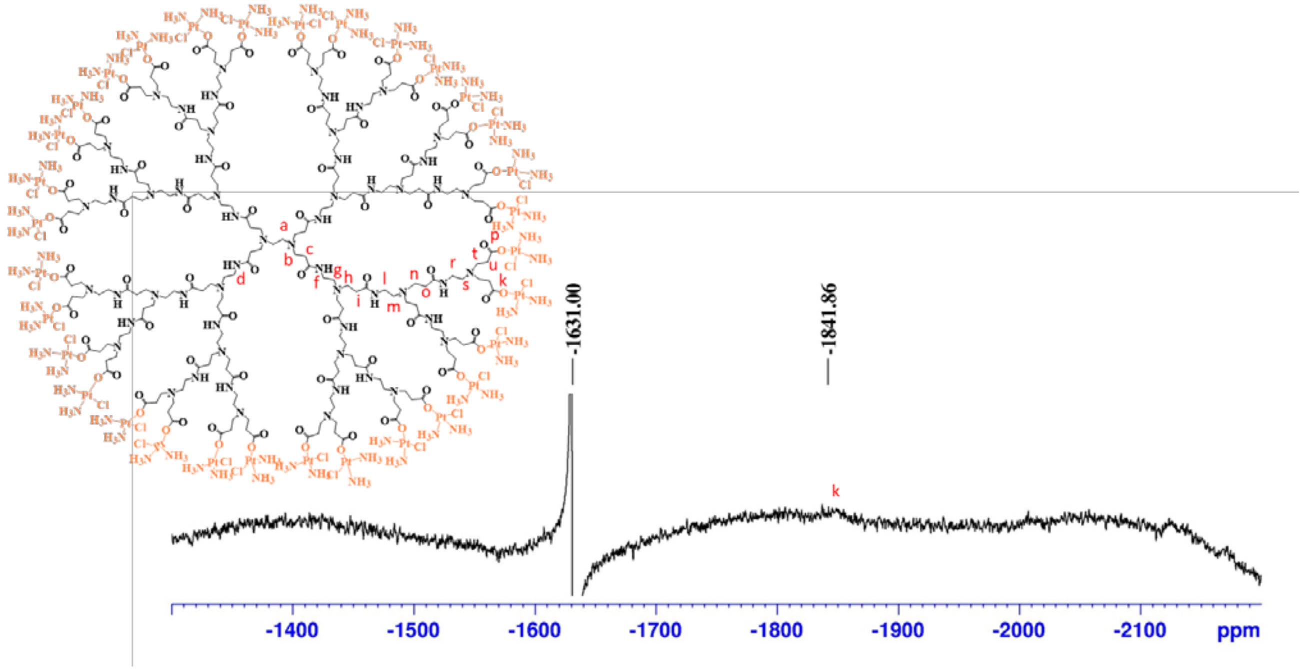

Figure S19: ^{195}Pt-NMR spectrum of monodentate G2.5(COOPt(NH_3)$_2$Cl)$_{32}$ in D_2O, with K_2PtCl_4 as an external reference (-1631 ppm).

Table S1: Molecular weight of the cisplatin-metallodendrimers in a monodentate form.

	**G0.5(COOPt(NH_3)$_2$Cl)$_8$**	**G1.5(COOPt(NH_3)$_2$Cl)$_{16}$**	**G2.5(COOPt(NH_3)$_2$Cl)$_{32}$**
**Molecular weight**	3201.90	6800.29	13997.08
**m/z calculated**	1606.68	1013.47	974.82
**m/z found**	1606.46<br>[M+2H$^+$+ H_2O]$^{2+}$<br><br>$C_{46}H_{118}Cl_8N_{26}O_{21}Pt_8^{2+}$	1013.46<br>[M+3H$^+$]$^{3+}$<br><br>$C_{110}H_{205}N_{30}O_{44}Pt_2{}^{3+}$	974.65<br>[M+H$^+$]$^+$<br><br>$C_{238}H_{562}Cl_{29}N_{116}O_{92}Pt_{29}^{+}$

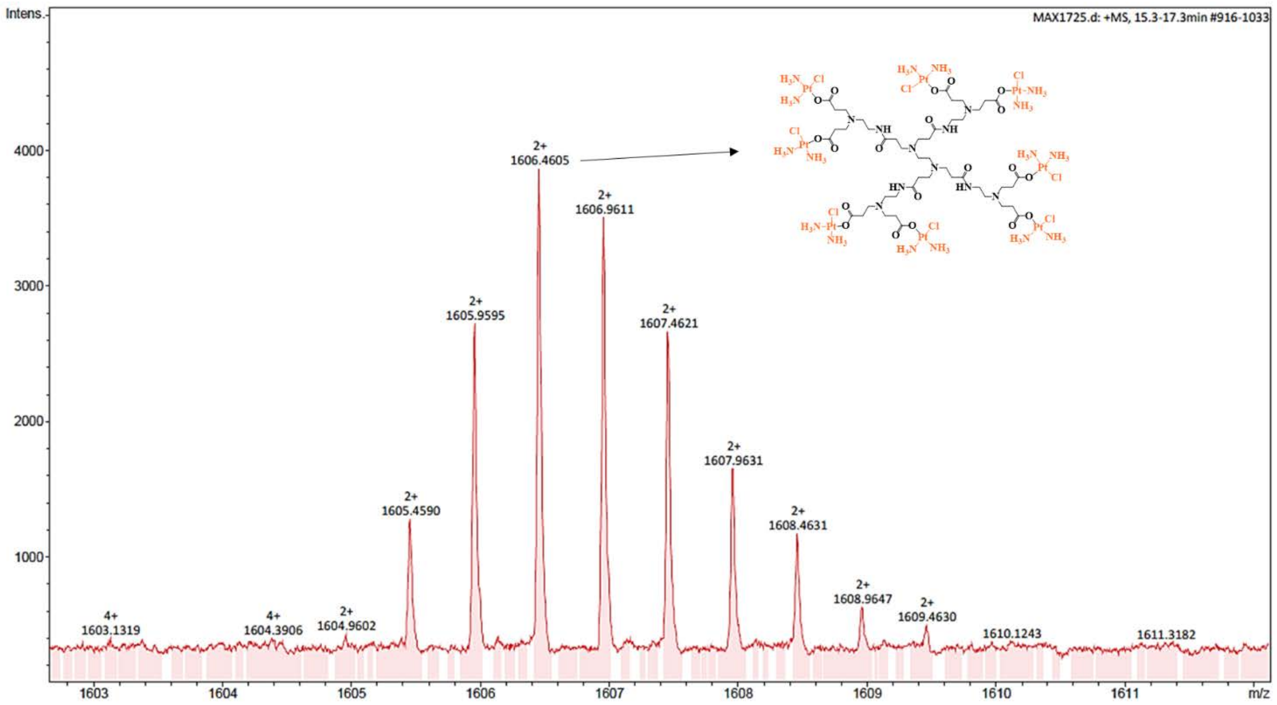

Figure S20: TOF-MS (ESI+) mass spectrum of monodentate G0.5(COOPt$(NH_3)_2$Cl)$_8$ metallodendrimer.

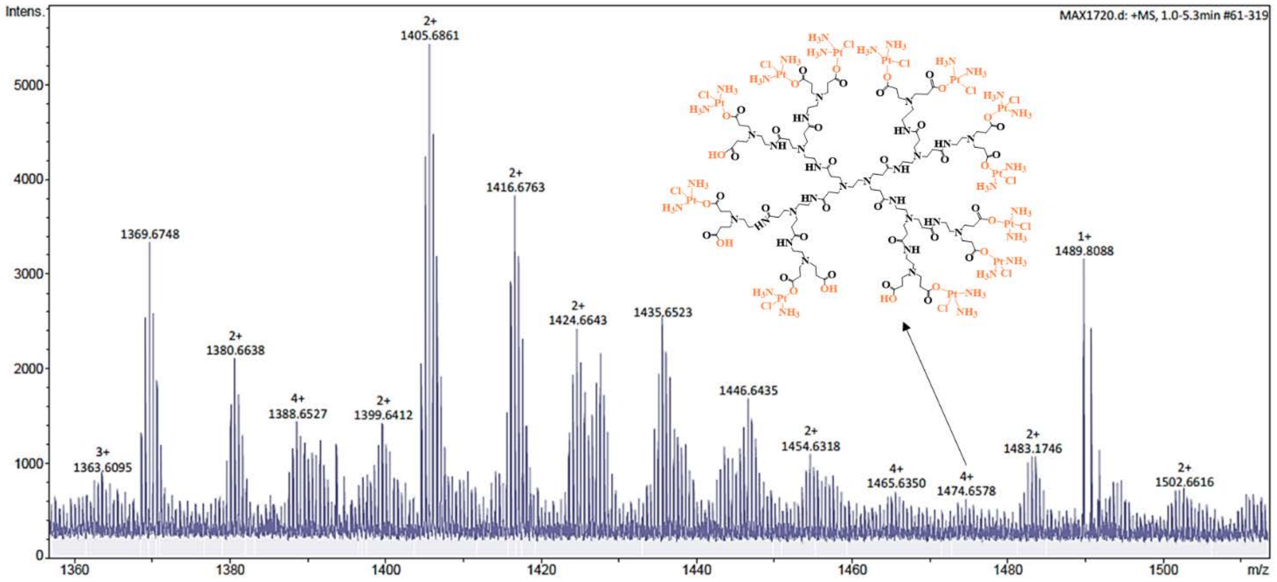

Figure S21: TOF-MS (ESI+) mass spectrum of monodentate G1.5(COOPt$(NH_3)_2$Cl)$_{16}$ metallodendrimer.

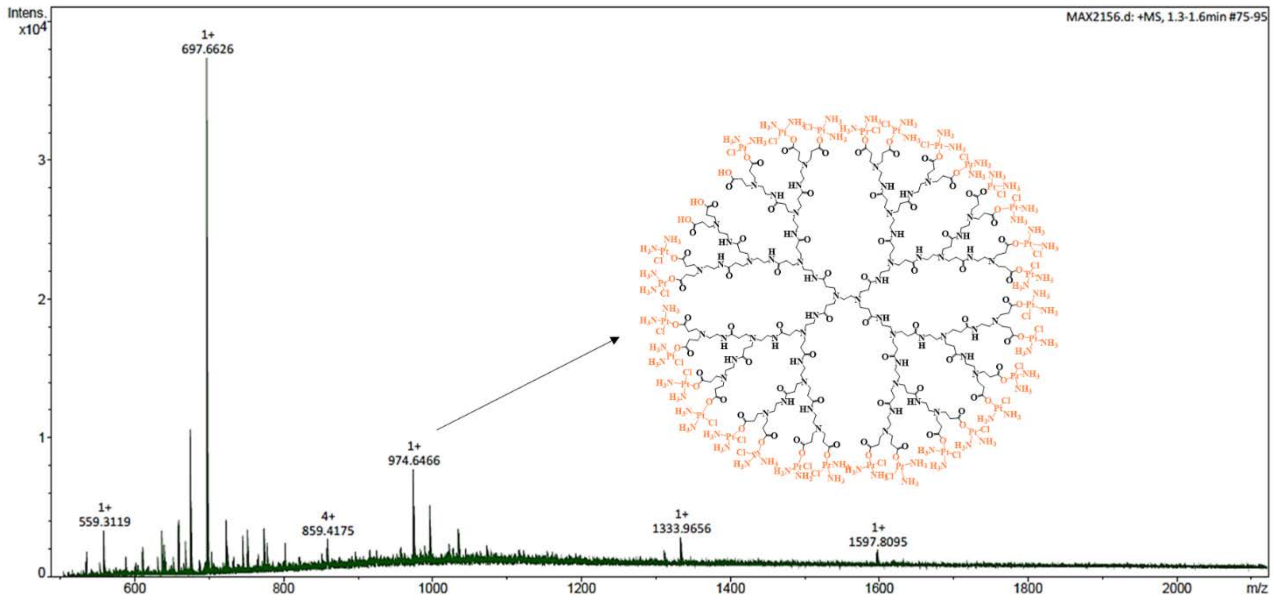

Figure S22: TOF-MS (ESI+) mass spectrum of monodentate G2.5(COOPt(NH_3)$_2$Cl)$_{32}$ metallodendrimer.

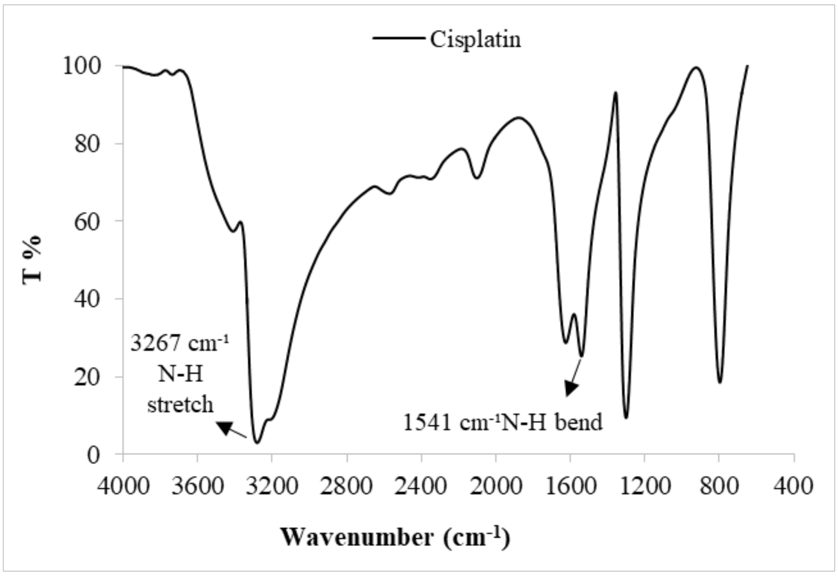

Figure S23: FTIR spectra of cisplatin in KBr pellet.

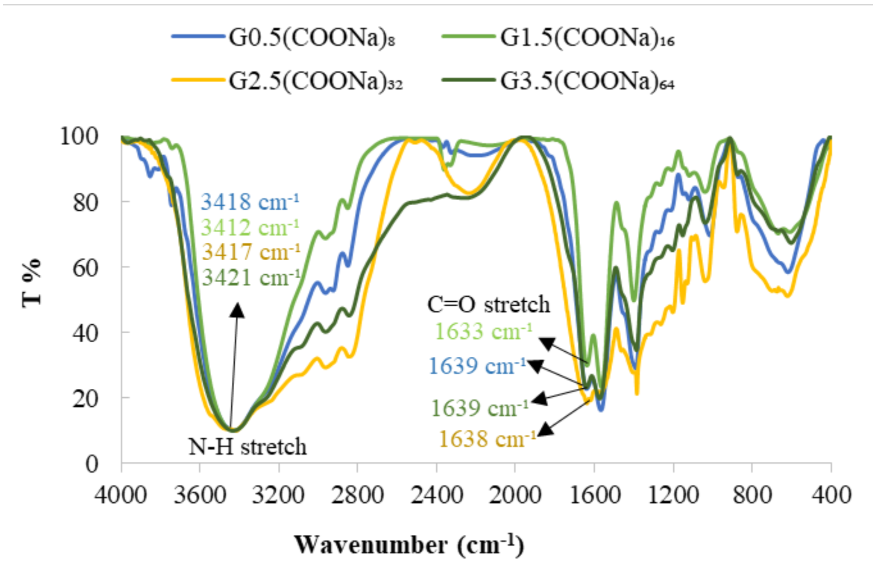

Figure S24: FTIR spectra of different generations of anionic PAMAM dendrimers (G0.5-G3.5) in KBr pellet.

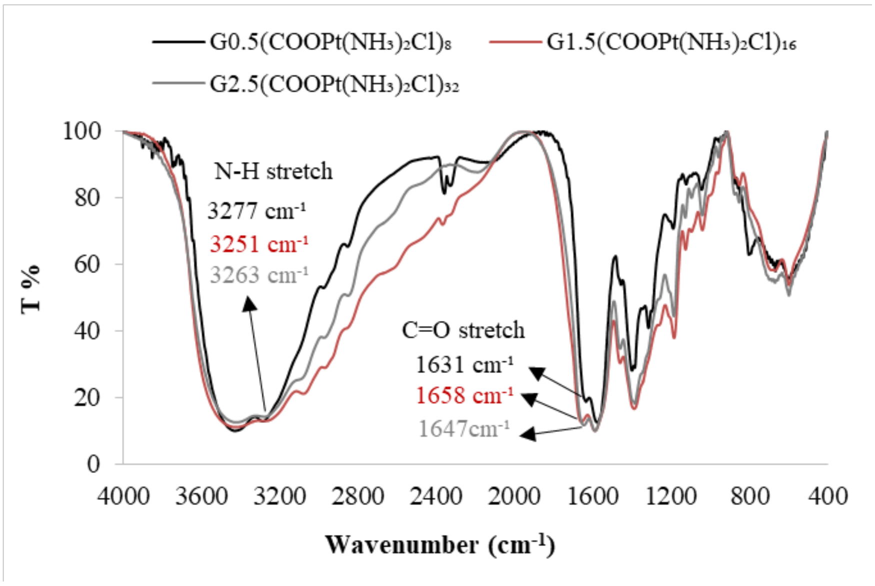

Figure S25: FTIR spectra of metallodendrimers conjugated with cisplatin in monodentate form. The spectra were performed in KBr pellet.

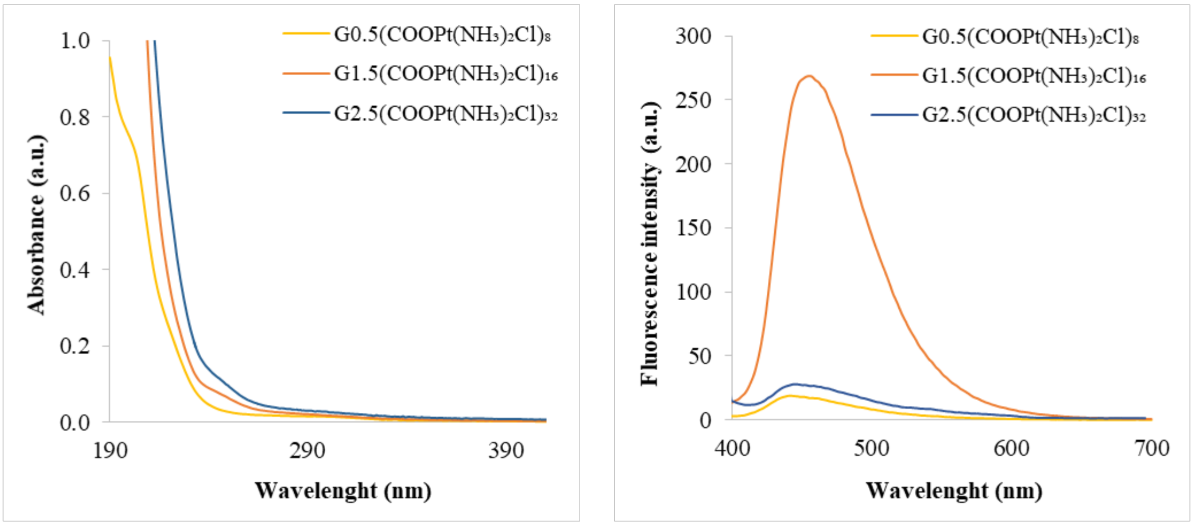

(a) (b)

Figure S26: a) Absorption spectra of cisplatin-metallodendrimers in the monodentate form recorded at a concentration of 40 μM in ultrapure water and b) Emission (λ_{ex} = 380 nm) spectra of cisplatin-metallodendrimers in the monodentate form recorded at a concentration of 500 μM in ultrapure water.

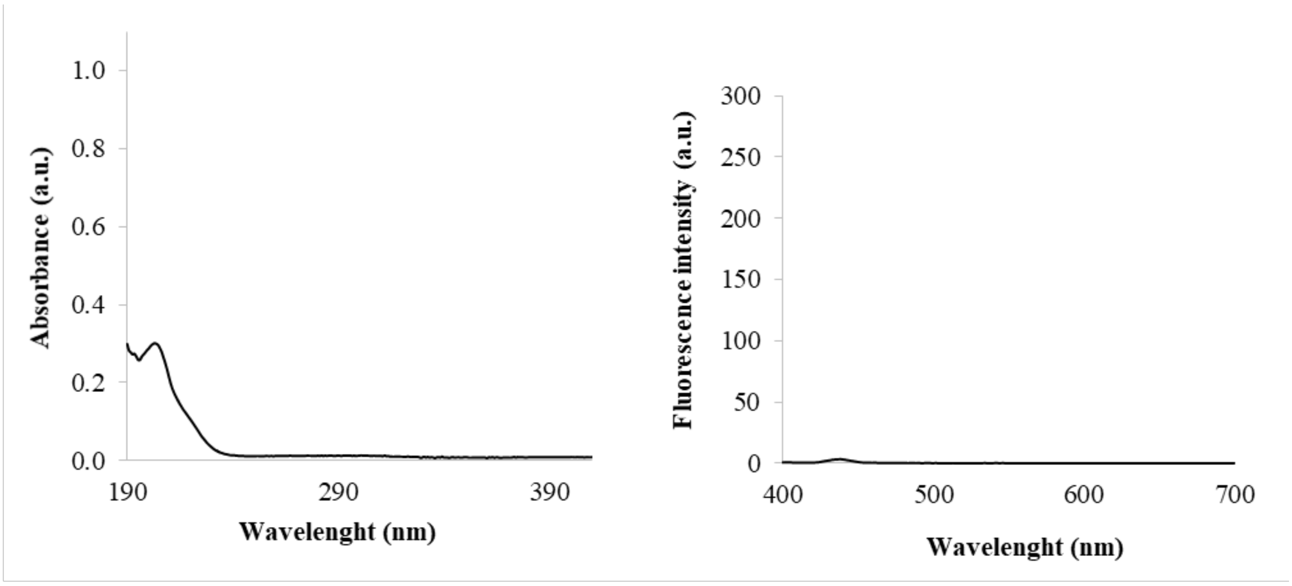

(a) (b)

Figure S27: a) Absorption spectra of cisplatin recorded at a concentration of 40 μM of in ultrapure water and b) Emission (λ_{ex} = 380 nm) spectra of cisplatin recorded at a concentration of 500 μM in ultrapure water.

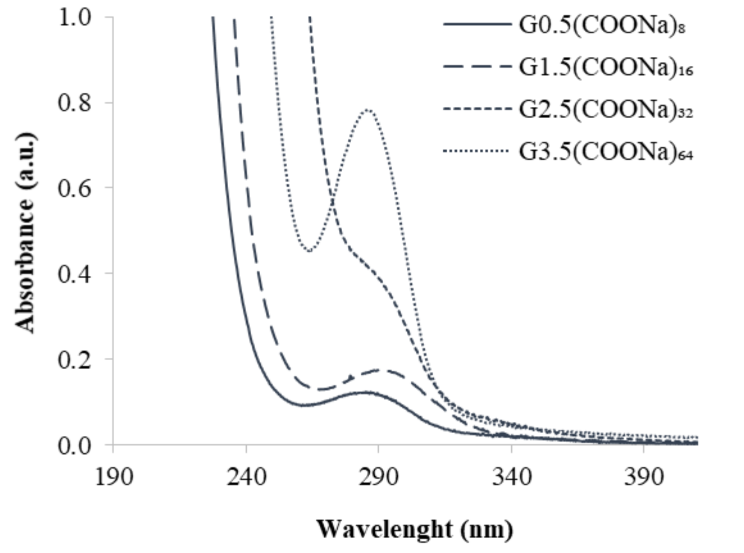

Figure S28: UV-Vis spectra of anionic PAMAM dendrimers at a concentration of 500 μM in ultrapure water.

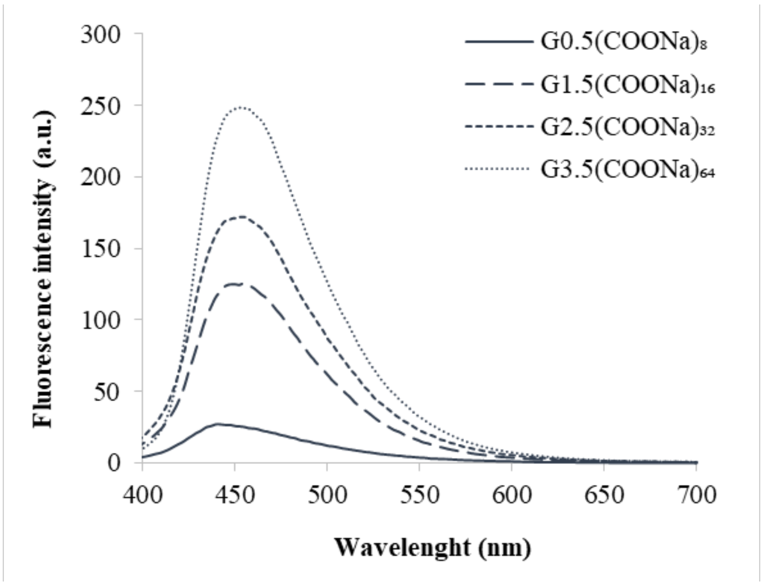

Figure S29: Emission (λ_{ex}= 380 nm) of anionic PAMAM dendrimers at a concentration of 500 μM in ultrapure water.

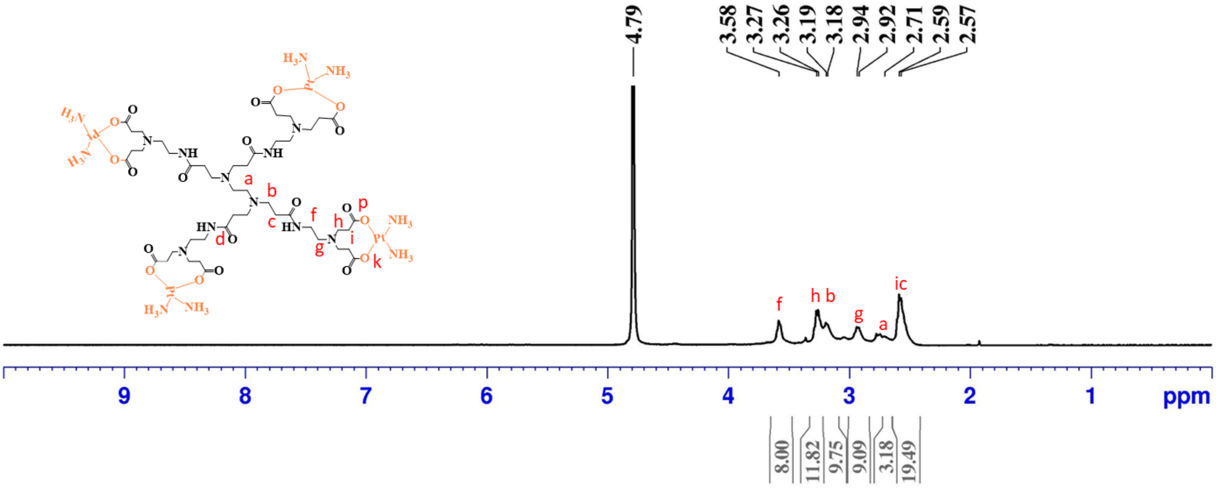

Figure S30: ¹H-NMR spectrum of bidentate G0.5(COOPt(NH3)2)4 in D2O.

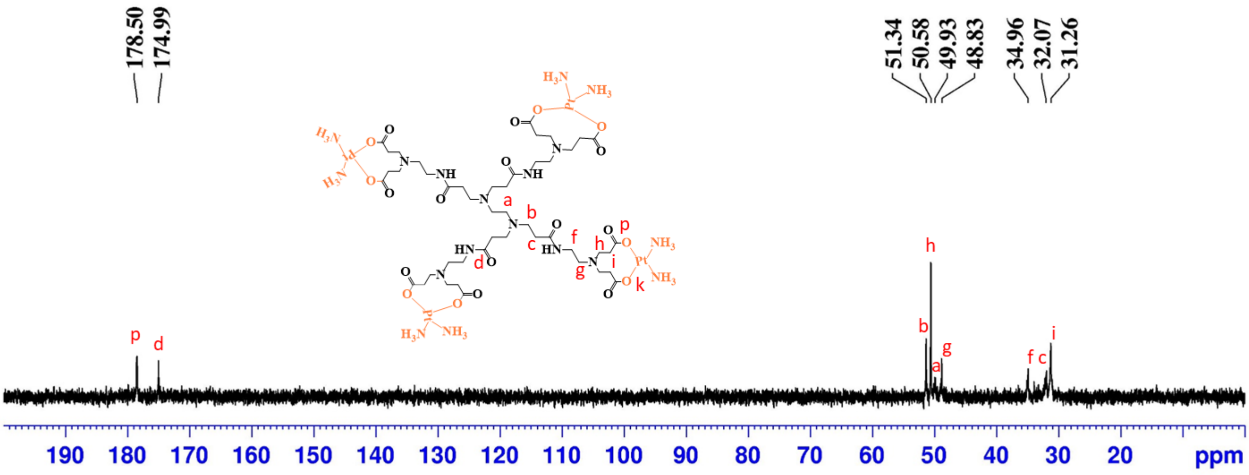

Figure S31: ¹³C-NMR spectrum of bidentate G0.5(COOPt(NH3)2)4 in D2O.

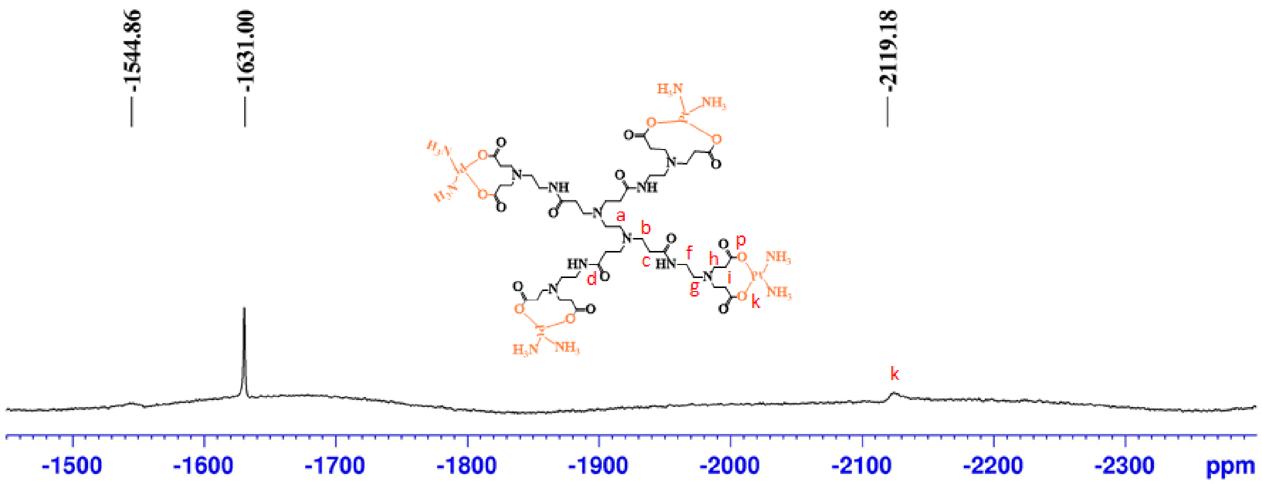

Figure S32: ^{195}Pt-NMR spectrum of bidentate G0.5COO(Pt(NH$_3$)$_2$)$_4$ in D$_2$O, with K$_2$PtCl$_4$ as external reference (-1631 ppm).

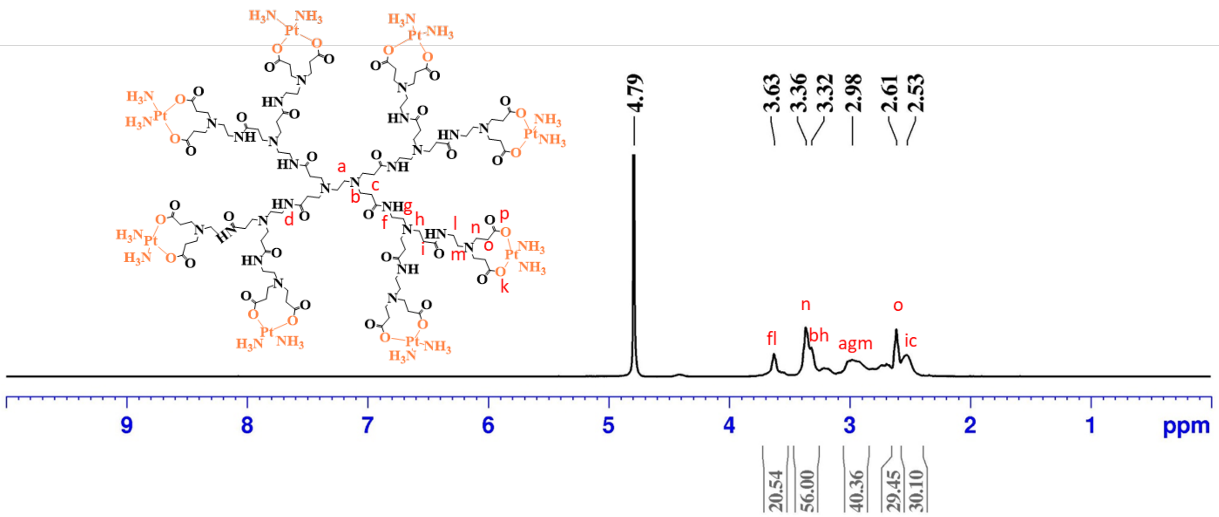

Figure S33: ^{1}H-NMR spectrum of bidentate G1.5COO(Pt(NH$_3$)$_2$)$_8$ in D$_2$O.

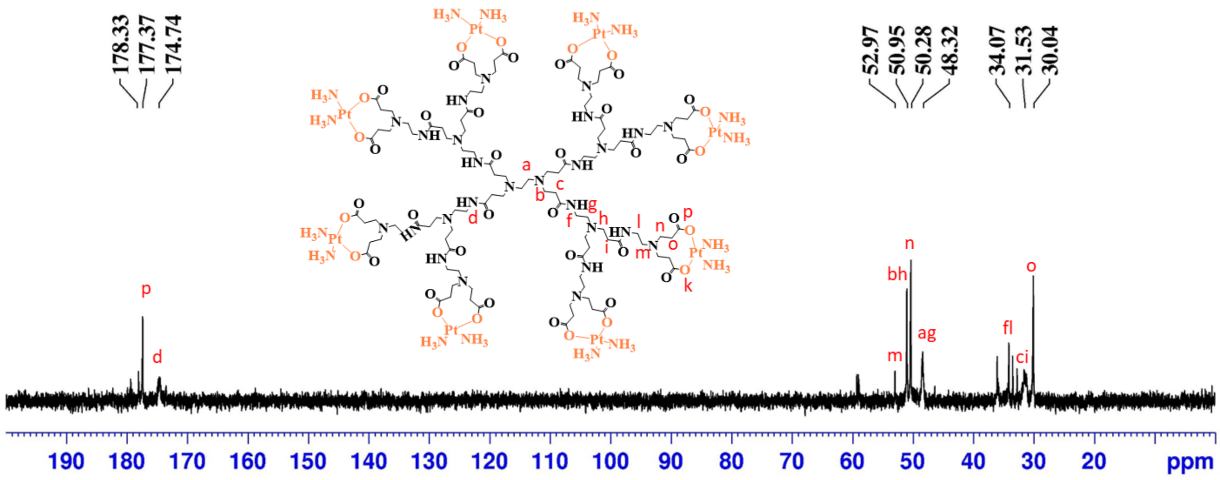

Figure S34: ^{13}C-NMR spectrum of bidentate G1.5COOPt(NH_3)$_2$)$_8$ in D_2O.

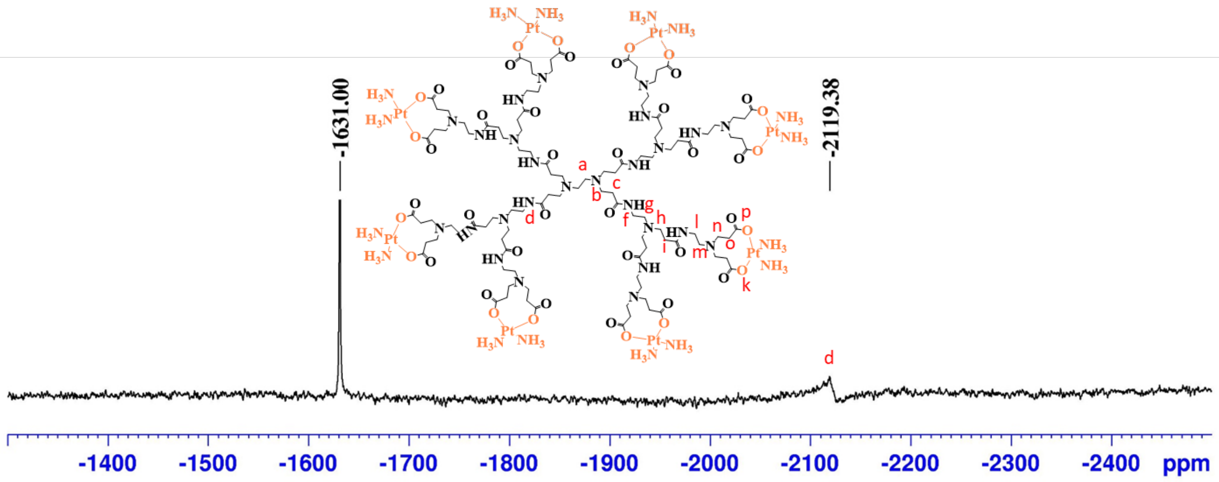

Figure S35: ^{195}Pt-NMR spectrum of bidentate G1.5COO(Pt(NH_3)$_2$)$_8$ in D_2O, with K_2PtCl_4 as external reference (-1631 ppm).

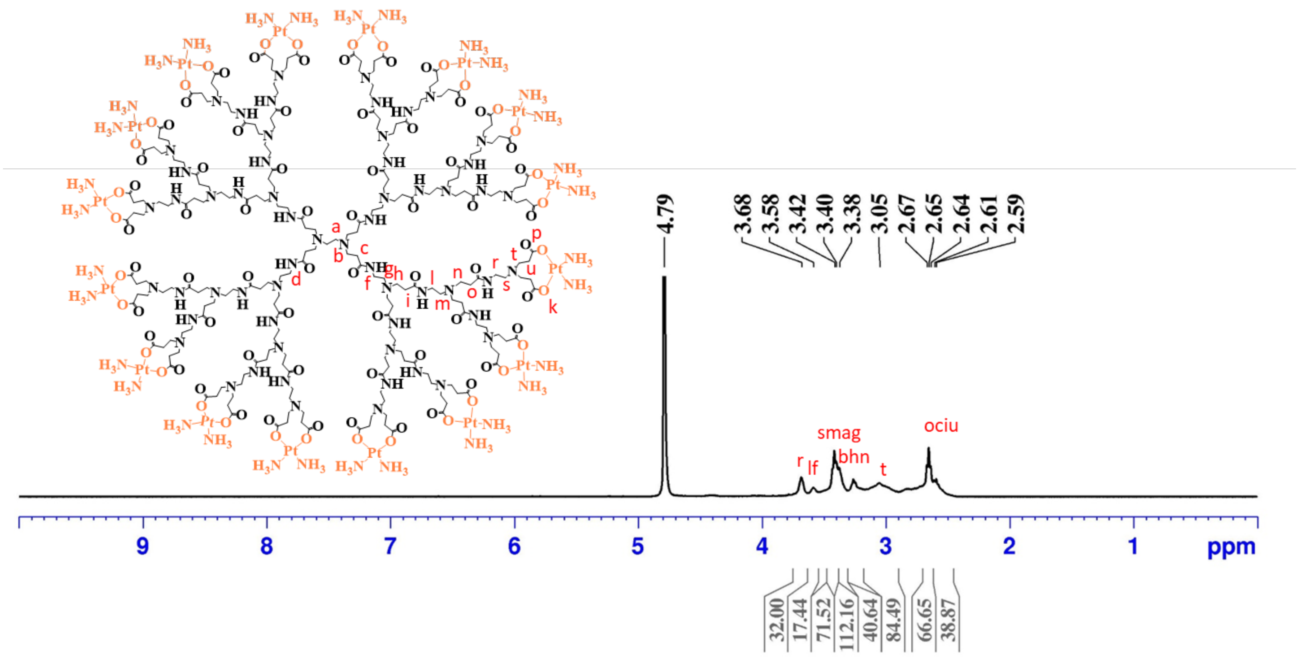

Figure S36: ^{1}H-NMR spectrum of bidentate G2.5COO(Pt(NH$_3$)$_2$)$_{16}$ in D$_2$O.

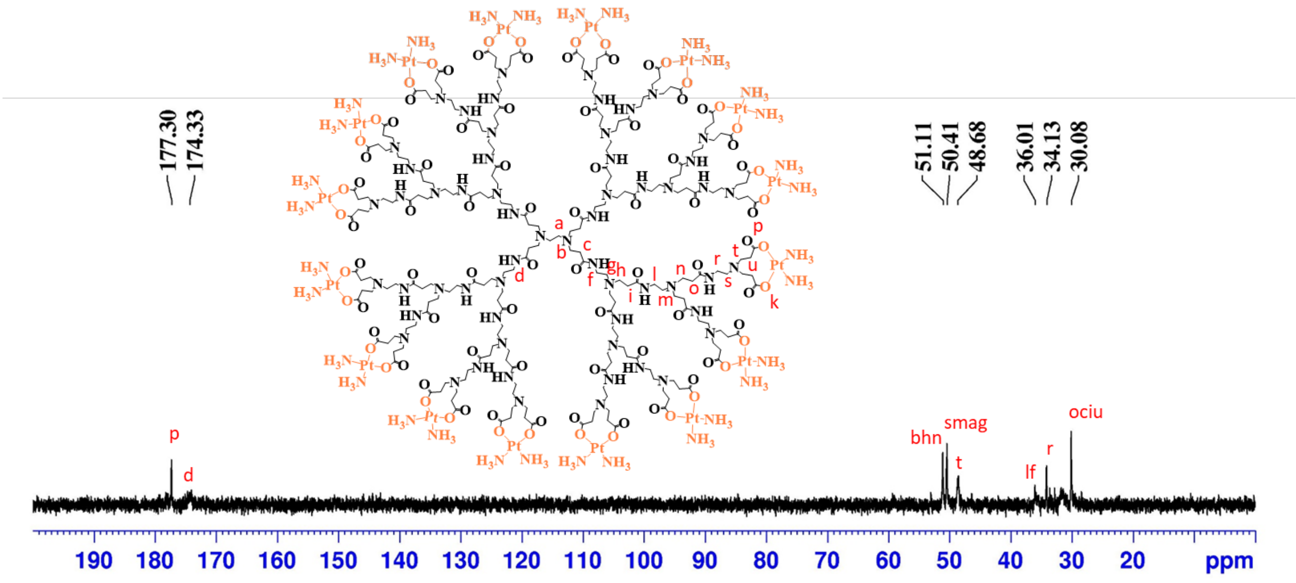

Figure S37: ^{13}C-NMR spectrum of bidentate G2.5COO(Pt(NH$_3$)$_2$)$_{16}$ in D$_2$O.

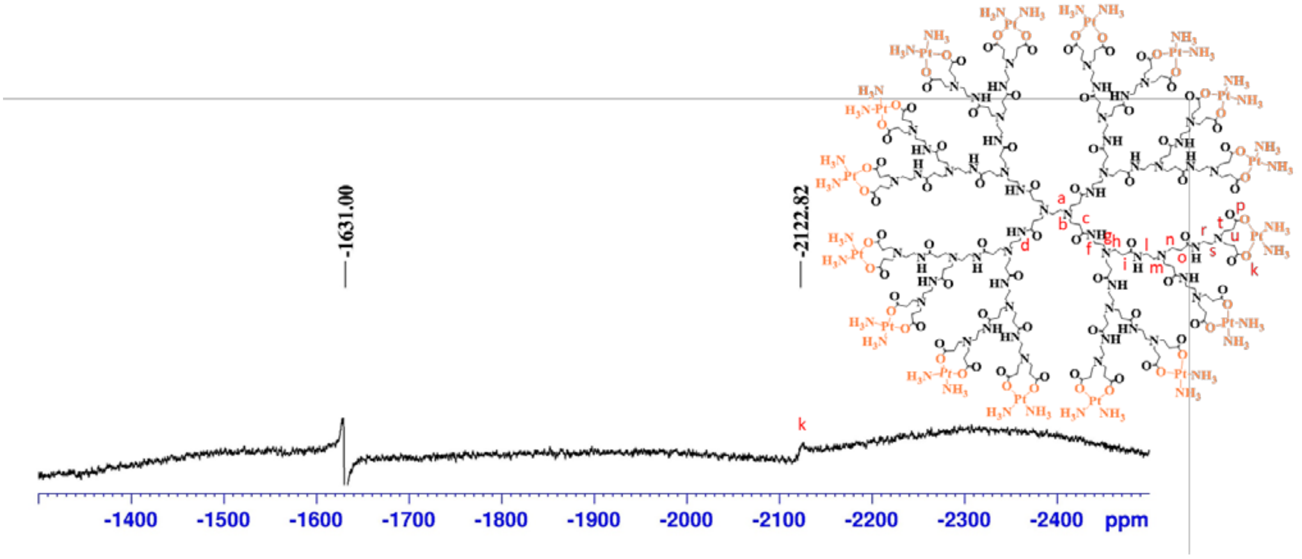

Figure S38: ^{195}Pt-NMR spectrum of bidentate G2.5COO($Pt(NH_3)_2$)$_{16}$ in D_2O, with K_2PtCl_4 as external reference (-1631 ppm).

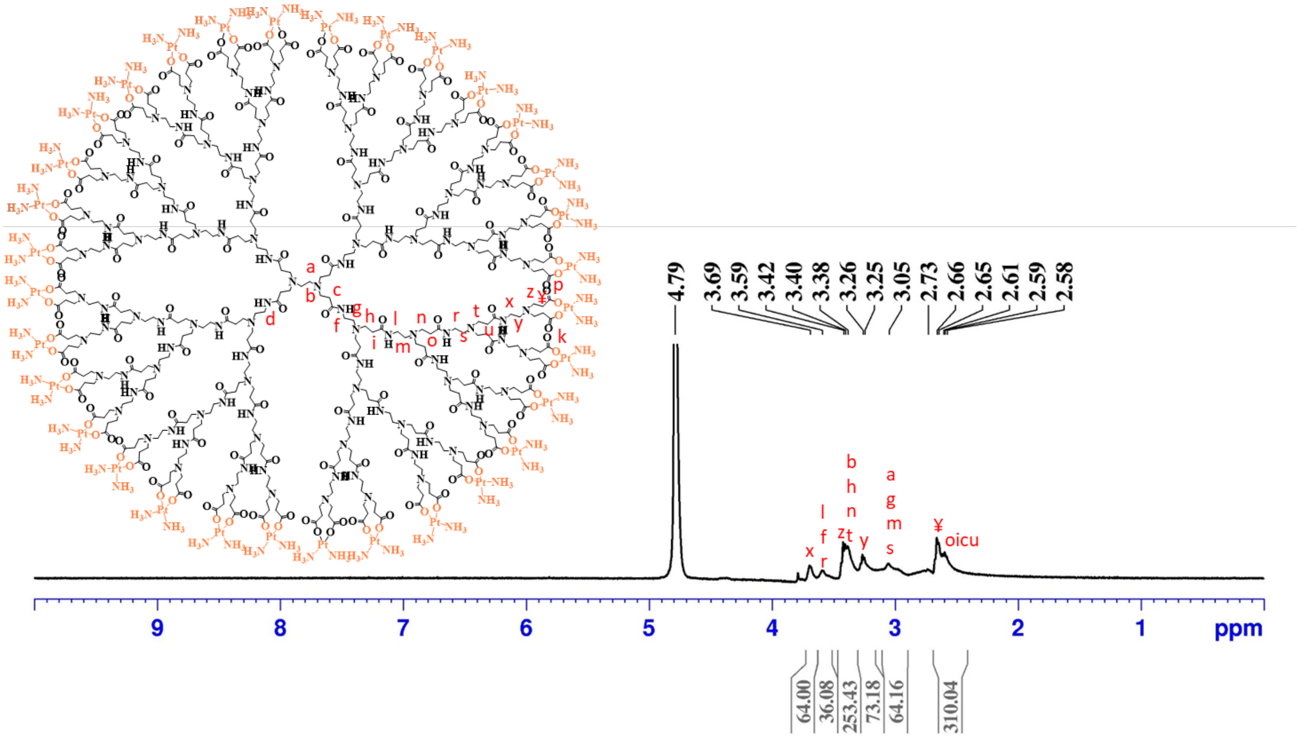

Figure S39: ^{1}H-NMR spectrum of bidentate G3.5COO($Pt(NH_3)_2$)$_{32}$ in D_2O.

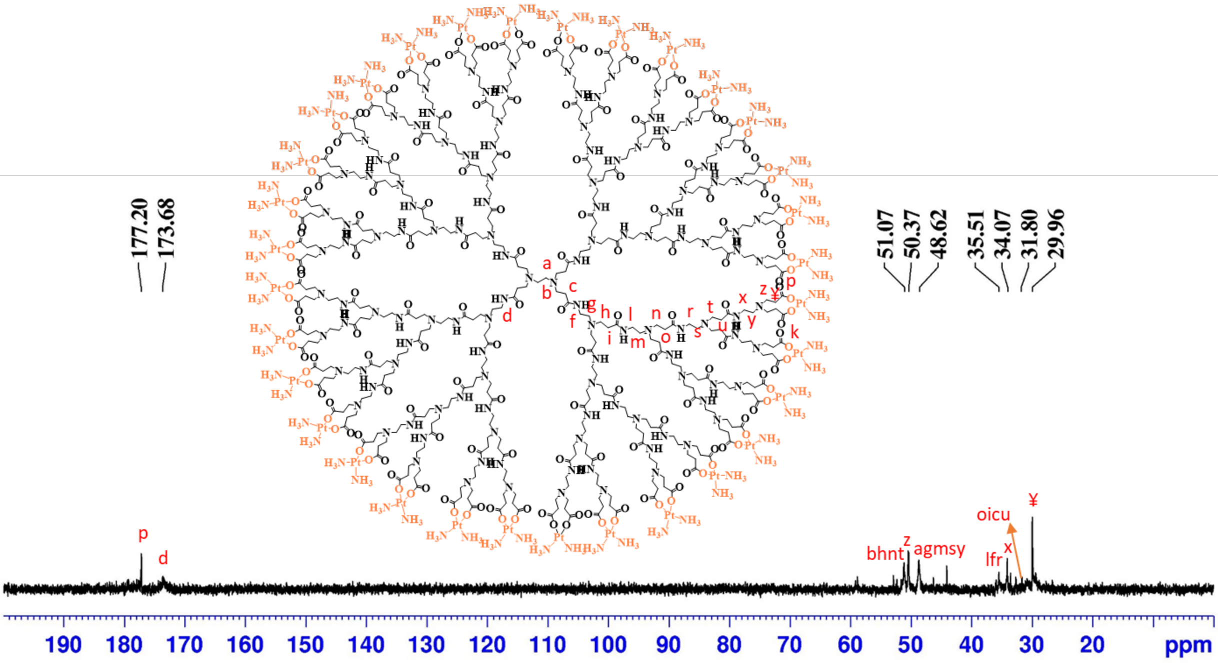

Figure S40: ^{13}C-NMR spectrum of bidentate G3.5COO$(Pt(NH_3)_2)_{32}$ in D_2O.

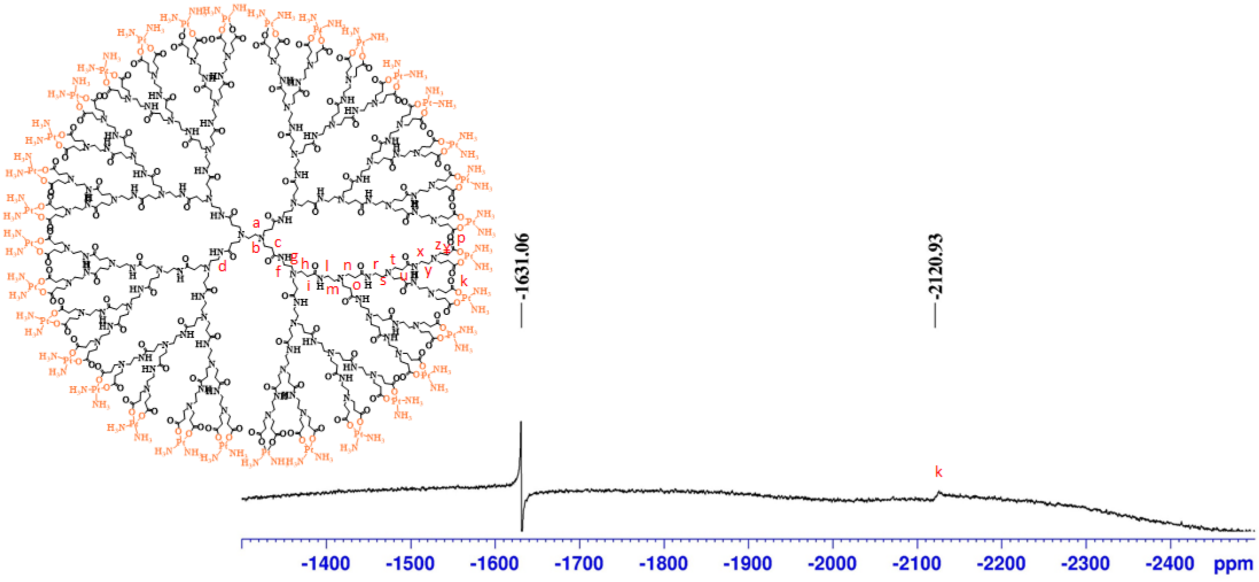

Figure S41: ^{195}Pt-NMR spectrum of bidentate G3.5COO$(Pt(NH_3)_2)_{32}$ in D_2O, with K_2PtCl_4 as external reference (-1631 ppm).

Table S2: Molecular weight of the cisplatin-metallodendrimers in a bidentate form.

	G0.5COO(Pt(NH3)2)4	G1.5COO(Pt(NH3)2)8	G2.5COO(Pt(NH3)2)16	G3.5COO(Pt(NH3)2)32
**Molecular weight**	2001.72	4399.92	9196.34	18789.17
**m/z calculated**	1023.27	1466.80	4598.51	974.82
**m/z found**	1023.27 $[M+2Na^+]^{2+}$ $C_{46}H_{96}N_{18}Na_2O_{20}Pt_4^{2+}$	1466.83 $[M+3H^+]^{3+}$ $C_{110}H_{227}N_{42}O_{44}Pt_8^{3+}$	4597.5 $[M+2H^+]^{2+}$ $C_{238}H_{482}N_{90}O_{92}Pt_{16}^{2+}$	974.64 $[M+H^+]^+$ $C_{494}H_{981}N_{180}O_{188}Pt_{29}^+$

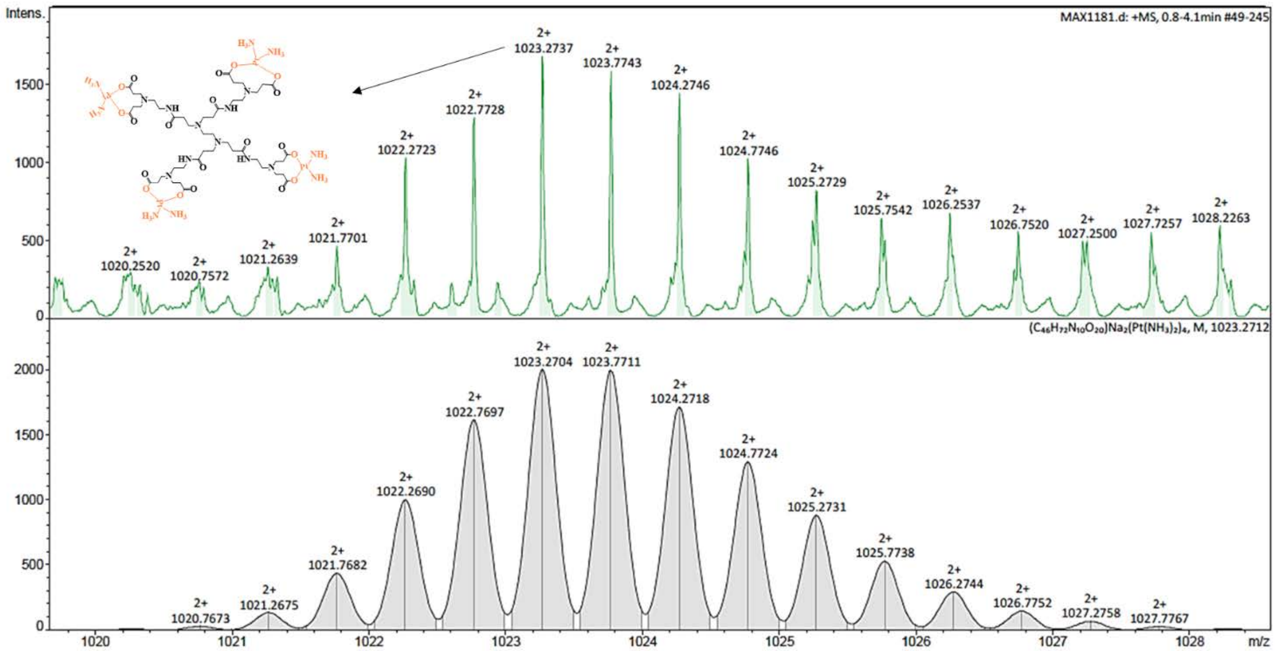

Figure S42: TOF-MS (ESI+) mass spectrum of bidentate G0.5COO(Pt(NH3)2)4 metallodendrimer.

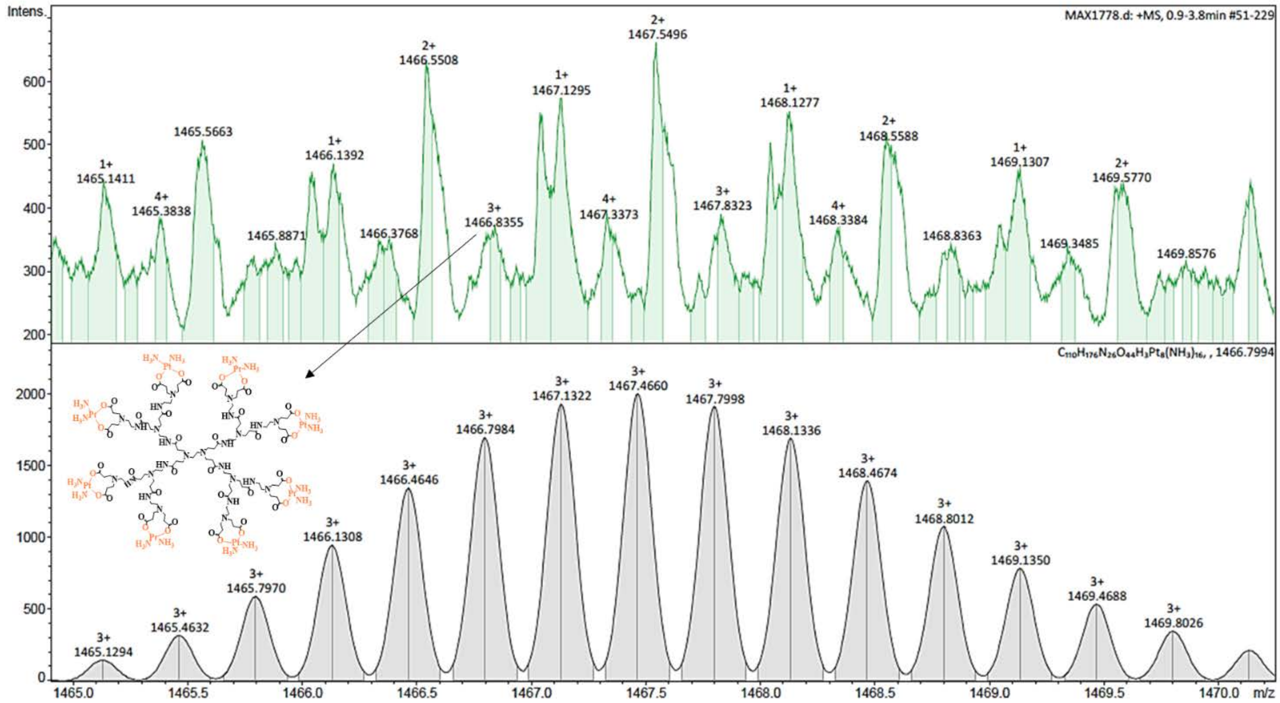

Figure S43: TOF-MS (ESI+) mass spectrum of bidentate G1.5COO(Pt(NH_3)$_2$)$_8$ metallodendrimer.

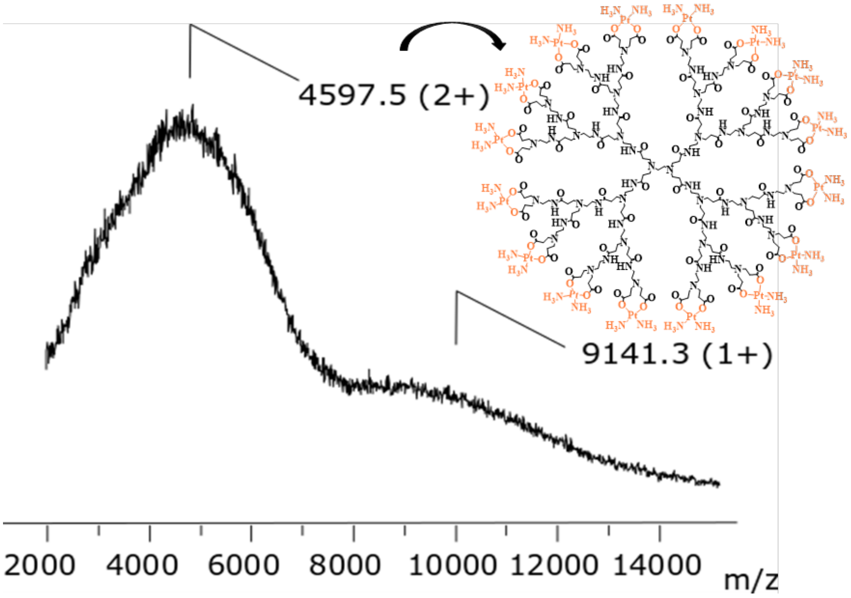

Figure S44: TOF-MS (MALDI) mass spectrum of bidentate G2.5COO(Pt(NH_3)$_2$)$_{16}$ metallodendrimer.

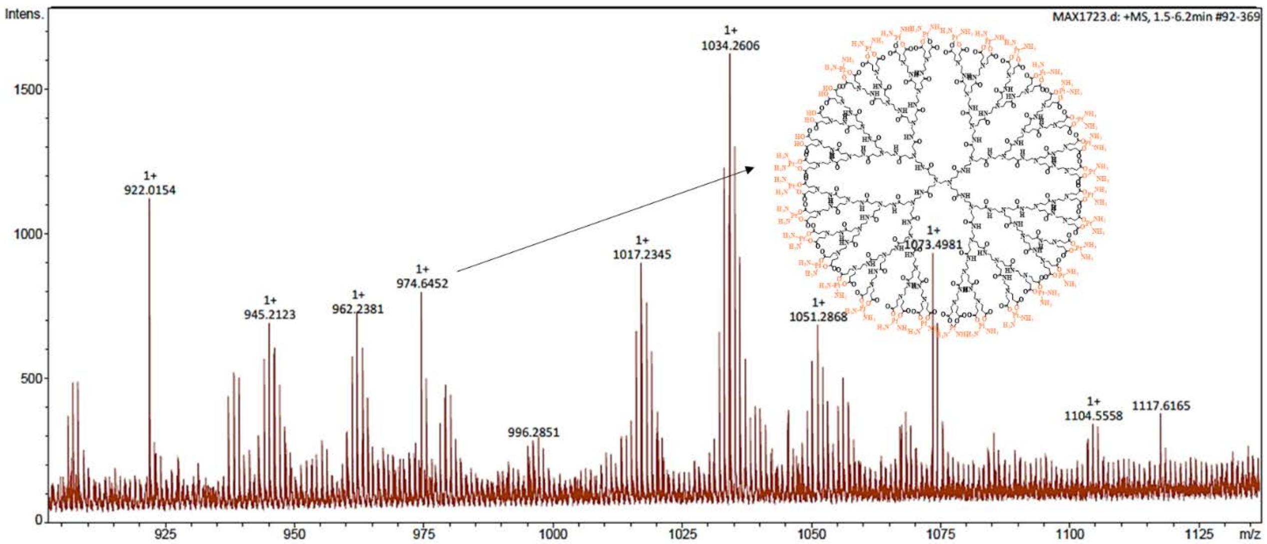

Figure S45: TOF-MS (ESI+) mass spectrum of bidentate G3.5COO(Pt(NH_3)$_2$)$_{32}$ metallodendrimer.

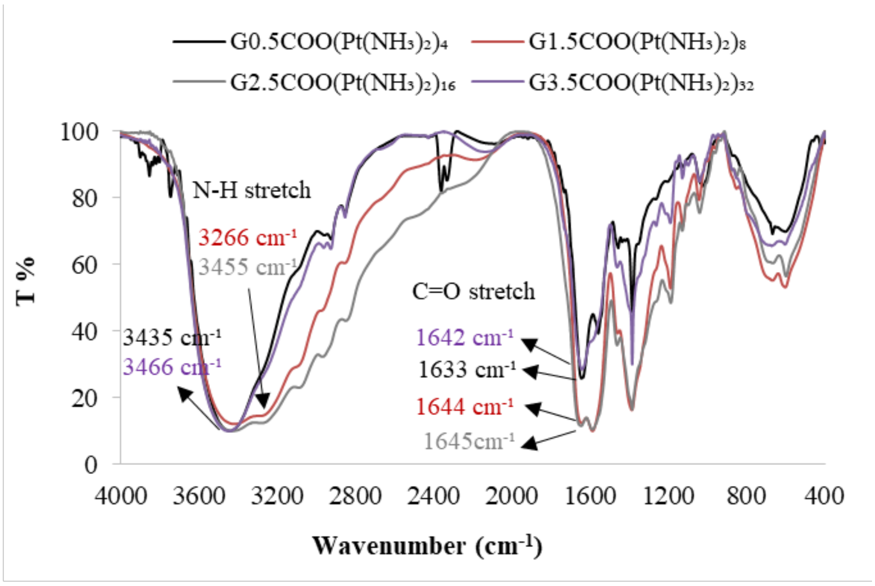

Figure S46: FTIR spectrum of metallodendrimers conjugated with cisplatin in bidentate form. The spectrum was performed in KBr pellet.

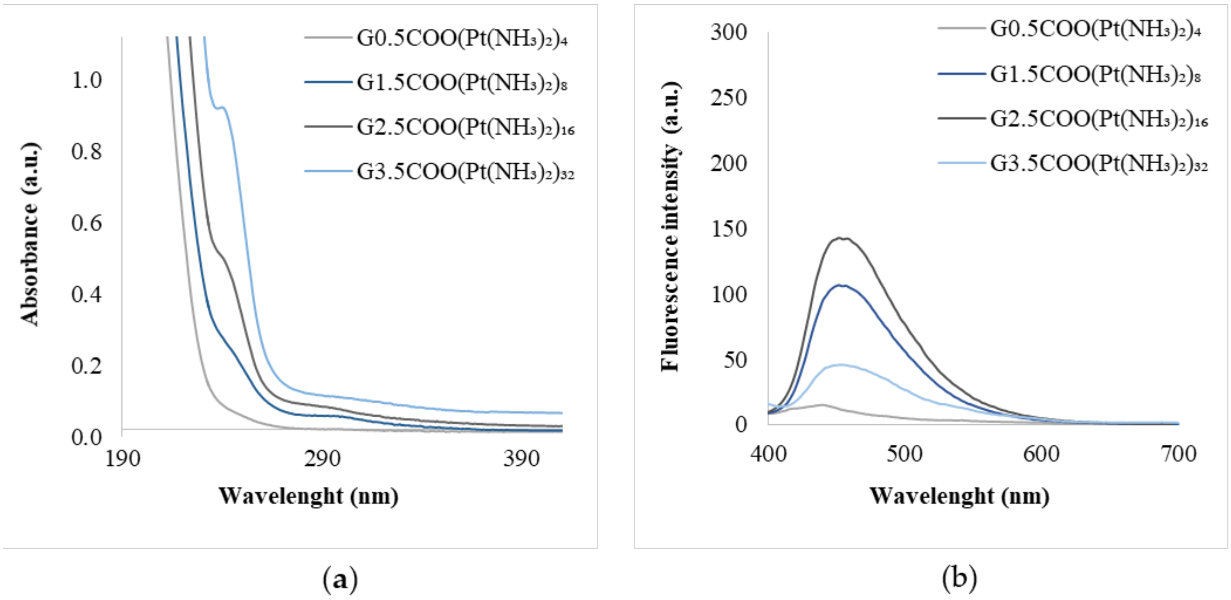

Figure S47: a) Absorption spectra of cisplatin-metallodendrimers in the bidentate form recorded at a concentration of 40 µM in ultrapure water and b) Emission (λ_{ex} = 380 nm) spectra of cisplatin-metallodendrimers in the bidentate form recorded at a concentration of 500 µM in ultrapure water.

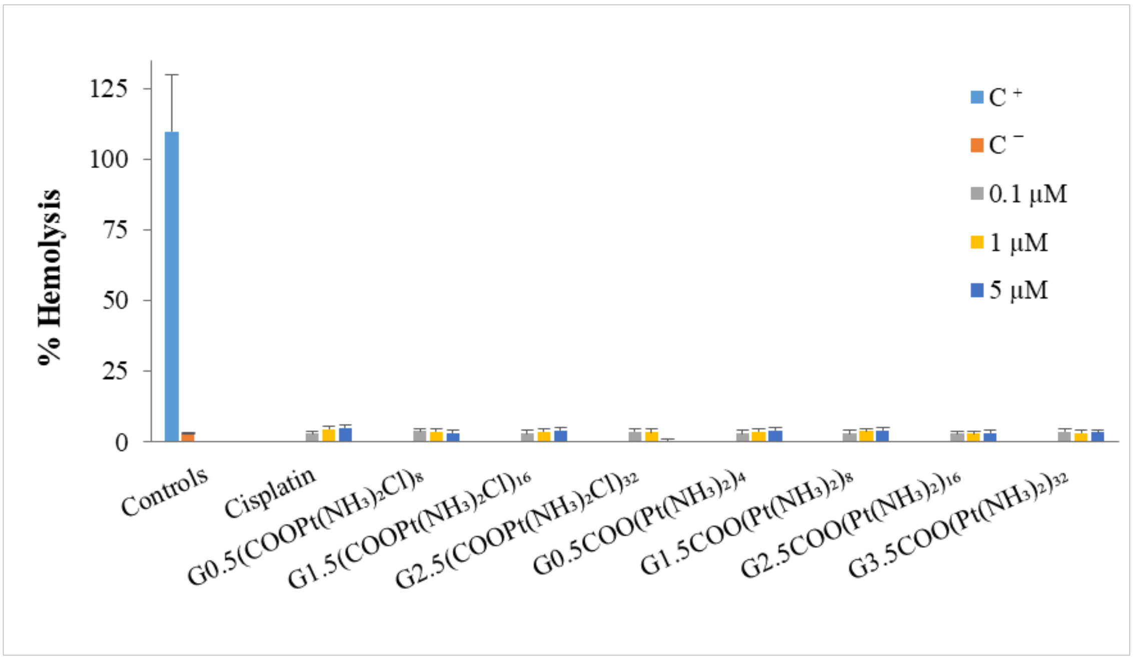

Figure S48: Hematoxicity of the free cisplatin and prepared cisplatin-metallodendrimers in healthy human blood. Blood was treated for 3 h with different concentrations (0.1, 1, and 5 µM) of the metallodendrimers and free cisplatin. The positive and negative control are represented by C^+ and C^-, respectively. The results are expressed as mean ± SD of at least three independent experiments performed in triplicate.

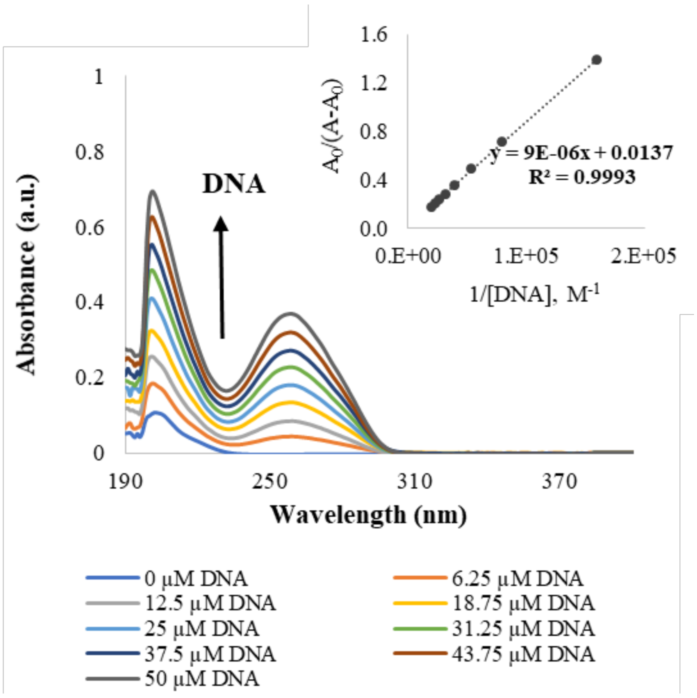

Figure S49: Representative UV-visible spectra of cisplatin with increasing concentration of CT-DNA (0, 6.25, 12.5, 18.75, 25, 31.25, 37.5, 43.75 and 50 µM) in 5 mM Tris-HCl/50 mM NaCl at pH 7.4. The inset corresponds to the plot of $A_0/(A-A_0)$ versus 1/[DNA], which is used to determine the binding constant. The arrow indicates the direction of increasing the concentration of DNA.
